# Effects of Lysolecithin on Growth Performance, Antioxidant Capacity, and Lipid Metabolism of *Litopenaeus vannamei*

**DOI:** 10.3390/antiox14101209

**Published:** 2025-10-06

**Authors:** Yun Wang, Hailiang Yan, Hong Liang, Yafei Duan, Jun Wang, Chuanpeng Zhou, Zhong Huang

**Affiliations:** 1 Key Laboratory of Aquatic Product Processing, Ministry of Agriculture and Rural Affairs, State Key Laboratory of Mariculture Biobreeding and Sustainable Goods, South China Sea Fisheries Research Institute, Chinese Academy of Fishery Sciences, Guangzhou 510300, China; hailiangyan813@163.com (H.Y.); lianghong99001122@163.com (H.L.); duanyafei@scsfri.ac.cn (Y.D.); jwang@scsfri.ac.cn (J.W.); zhoucp@scsfri.ac.cn (C.Z.); 2 Key Laboratory of Efficient Utilization and Processing of Marine Fishery Resources of Hainan Province, Hainan Engineering Research Center of Deep-Sea Aquaculture and Processing, Sanya Tropical Fisheries Research Institute, Sanya 572018, China; 3 Guangdong Provincial Key Laboratory of Fishery Ecology and Environment, South China Sea Fisheries Research Institute, Chinese Academy of Fisheries Sciences, Guangzhou 510300, China; 4 Shenzhen Base of South China Sea Fisheries Research Institute, Chinese Academy of Fishery Sciences, Shenzhen 518121, China; huangzhongnhs@163.com

**Keywords:** lysolecithins, growth, antioxidant capacity, lipid metabolism, oxidative stress, *Litopenaeus vannamei*

## Abstract

Lysolecithin, characterized by its superior emulsifying and stabilizing properties, facilitates nutrient absorption and is extensively utilized in aquatic feed formulation. Nevertheless, its precise function in shrimp nutrition and physiology remains inadequately understood. This study aimed to evaluate the feasibility and optimal dosage of replacing 2% soybean lecithin with varying levels of soybean lysolecithin (0–2%) in the diet of *Litopenaeus vannamei*. Growth performance, antioxidant indices, and lipid metabolism were assessed. The results demonstrated that dietary supplementation with 0.1% lysolecithin had the best growth performance, significantly improved lipid retention and apparent crude fat digestibility, while reducing malondialdehyde (MDA) levels in the hepatopancreas and alleviating endoplasmic reticulum (ER) stress. The 0.1% group also exhibited better hepatopancreatic tissue structure and lipid metabolic homeostasis. In contrast, higher inclusion levels (≥1.5%) led to increased lipid accumulation and enhanced activities of lipid metabolic enzymes but were associated with a risk of oxidative stress and less favorable tissue morphology. No significant differences in antioxidant enzyme activities were observed among groups. It is hypothesized that lysolecithin may regulate lipid metabolism and homeostasis via the Ca^2+^/CaMKKβ/AMPK signaling pathway; further studies are required to confirm this mechanism. In conclusion, 0.1% soybean lysolecithin is recommended as the optimal dietary level for *L. vannamei*, supporting its feasibility as a substitute for 2% soybean lecithin in shrimp feed.

## 1. Introduction

Oxidative stress, defined by an imbalance between the production of reactive oxygen species (ROS) and the organism’s antioxidant defense systems, constitutes a critical biological challenge faced by nearly all aerobic organisms [1]. The hepatopancreas of crustaceans, a pivotal organ for metabolism and detoxification, is especially vulnerable to oxidative damage due to its elevated metabolic activity and lipid turnover rates [2]. Such damage, frequently evidenced by lipid peroxidation, can compromise cellular membranes, disrupt metabolic homeostasis, and induce endoplasmic reticulum (ER) stress, ultimately jeopardizing growth and survival [3,4,5].

Soy lecithin are natural phospholipids obtained from soy and is widely used as nutritional supplements and emulsifiers in animal feed. Lysolecithin is produced via the enzymatic hydrolysis of lecithin by phospholipases, exhibiting stronger emulsifying than conventional lecithin due to the removal of one fatty acid chain [6]. Owing to the superior emulsifying stability and resistance to high temperature, lysolecithin can be used in lower quantities than lecithin. The increased hydrophilicity enhances their efficacy as emulsifiers, enabling more efficient formation of micelles of fatty acids, improving the efficiency of fat absorption and utilization, and leading to reduced leaching of water-soluble nutrients [7]. Lysolecithins have been extensively studied in animal nutrition. For instance, dietary supplementation with lysolecithins accelerates nutrient absorption, enhances nutrient digestibility and growth in livestock, including chickens and pigs, reduces fat deposition, and improves intestinal morphology [8,9]. Recent studies on lysolecithins in aquatic animals have demonstrated that lysolecithins can enhance aquatic animals’ growth, reduce the lipid demand of aquatic animals to improve the lipid utilization, modulate hepatic lipid metabolism as well as the antioxidant capacity of aquatic animals, and produce good effects on the health of aquatic animals [10,11,12,13]. These effects are potentially associated with the activation of energy-sensing pathways, such as AMP-activated protein kinase (AMPK), which regulates fatty acid β-oxidation and mitochondrial biogenesis, consequently decreasing the substrate availability for lipid peroxidation [14,15]. Moreover, by facilitating efficient lipid utilization, lysolecithin may help to alleviate metabolic overload in the endoplasmic reticulum (ER), a critical organelle involved in lipid synthesis and protein folding [16]. This, in turn, may reduce ER stress and its adverse effects. The present study was designed to assess the effects of replacing 2% soybean lecithin with varying levels of soybean lysolecithin (0–2%) on the growth performance, antioxidant activity, and lipid metabolism of *Litopenaeus vannamei*. By comprehensively evaluating these indicators, we aimed to determine both the feasibility and the optimal dosage of lysolecithin as a substitute for soybean lecithin in shrimp diets.

*Litopenaeus vannamei* is an important economic aquaculture species [17]. Due to its rapid growth, high meat yield, and strong adaptability, it has become a dominant species in global aquaculture [18]. In 2023, the worldwide production of *L. vannamei* in aquaculture surpassed 4.5 million tons, with China contributing 2.2 million tons [19]. This growth has established it as a major aquaculture product in numerous developing countries and economically disadvantaged regions, thereby significantly contributing to the enhancement of farmers’ incomes and the assurance of food security [20]. Consequently, lysolecithins have significant potential for application as feed additives.

However, there is limited information regarding the effect of lysolecithin supplementation on *L. vannamei* in crustacean studies. Herein, we investigated the effects of lysolecithin on growth performance, nutrient digestibility, antioxidant capacity, hepatopancreas morphology, and lipid metabolism of *L. vannamei*. This study utilizes a systematic and multi-tiered research design, implementing a comprehensive investigative strategy that encompasses multiple levels of analysis. It ranges from examining animal phenotypes, such as growth performance, to exploring molecular mechanisms, including antioxidant capacity and lipid metabolism. Additionally, it incorporates biochemical analyses of antioxidant indicators and extends to omics studies focused on lipid metabolomics. The multifaceted approach substantially augments the depth and scientific rigor of the research. The results will help figure out the mechanism of lysolecithin enhancing lipid utilization in *L. vannamei*.

## 2. Materials and Methods

### 2.1. Experimental Materials

The feedstuffs were obtained from Shandong Fuzhou Feng Agricultural Technology Co., Ltd. (Heze, China). Lysolecithin was sourced from the local supplier. Seven experimental diets were formulated, comprising a positive control group (2.0% of soy lecithin, DL2), six diets supplemented with various lysolecithin levels: 0% (RL0), 0.1% (RL0.1), 0.5% (RL0.5), 1% (RL1), 1.5% (RL1.5), and 2% (RL2). The formulation and proximate composition of the seven experimental diets are shown in Table 1. The diets were formulated following the procedure described by Yan [21]. Y_2_O_3_ was used as an inert marker at a concentration of 0.01% in diets. Four replicates were set for each group. All ingredients were ground and passed through an 80-mesh sieve, subsequently mixed completely with oil and water. The mixture was extruded using a twin-screw extruder (F-26, South China University of Technology, Guangzhou, China) to form long strands with diameters of 1.0 and 1.5 mm. The strands were processed into pellet feed using a pelletizer (G-500, South China University of Technology, Guangzhou, China). The pellet feed was kept at 90 °C in an oven for 90 min, followed by air-drying to ensure the moisture was less than 10%. All the feeds were stored at −20 °C until use.

### 2.2. Experimental Animals and Husbandry Management

Shrimp were temporarily housed at the Shenzhen base of the South China Sea Fisheries Research Institute, Chinese Academy of Fishery Sciences. Healthy shrimp with an average body weight of were selected. A total of 840 healthy shrimp with an initial body weight of 2.22 ± 0.11 g were randomly allocated to 28 fiberglass tanks (500 L, bottle area 0.5 m^2^), with four tanks per group, and 30 shrimp per tank. During the experiment, the water temperature, salinity, pH, and dissolved oxygen were monitored and maintained at 29.8 ± 1.4 °C, 29.4 ± 0.8, 8.0 ± 0.1, and > 6.0 mg/L, respectively, during the whole feeding period. Shrimp were fed diets at 2–4% of the total initial body weight (IBW) per tank, three times daily at 8:00, 17:00, and 22:00. A feeding tray was placed into each tank. The remaining feed in the tray was recorded after one-hour post-feeding. The feeding quantity was modified according to weather conditions and feeding behavior. The feed consumption, death of shrimp was recorded to calculate the feed conversion (FCR) rate and survival rate (SR). The feeding trial lasted for 56 days.

### 2.3. Sample Collection and Storage

Shrimp feces were collected using a siphoning method from the fifth week of farming. Two hours post-feeding, fresh, intact feces with a coating were selected using tweezers, washed with distilled water, dried with filter paper, and subsequently stored in 50 mL white plastic bottles at −20 °C for future use.

Shrimp were fasted for 24 h at the end of the feeding trial. Shrimp number and weight were recorded per tank. Five shrimp with similar weights were randomly taken from each tank for nutritional composition analysis. All of the samples, including whole shrimp, muscle, and hepatopancreas, were stored at −20 °C. Ten shrimp were randomly selected from each tank. The cephalothorax surface was cleansed with gauze to eliminate seawater, and the shrimp were subsequently wiped with 75% ethanol-soaked cotton balls. The hepatopancreas was sampled and rapidly frozen in liquid nitrogen, followed by storage at −80 °C. The hepatopancreas from another two shrimp was fixed using Davison’s solution for morphological examination. Five shrimp were selected per tank for fatty acid composition analysis of hepatopancreas and muscle. For lipidomics analysis, hepatopancreas was collected from four shrimp from each tank, and was rapidly frozen in liquid nitrogen before being stored at −80 °C.

### 2.4. Growth Parameters Analysis

Fecal and feed samples were sent to Standard Testing Co., Ltd. (Qingdao, China) for Y_2_O_3_ mass fraction analysis. Equations were used to calculate growth parameters for assessing diet quality as follows:

Survival (%) = final number of shrimp/initial number of shrimp × 100(1)

Weight gain (WG, %) = (W*_t_* − W*_i_*)/W*_i_* × 100 (2)

Specific growth rate (SGR, % day^−1^) = 100 × (Ln W*_t_* − Ln W*_i_*)/*t* (3)

Feed conversion ratio (FCR) = W*_f_*/(W*_t_* − W*_i_*) (4)

Protein retention (PR, %) = 100 × retained protein (g)/protein fed (g)(5)

Lipid retention (LR, %) = 100 × retained lipid (g)/lipid fed (g)(6)

where W*_t_*, represents the average final body weight (g) at feeding time *t* (days), W*_i_* is the average initial body weight (g), W*_f_* is the total feed consumption as dry matter (g).

Apparent digestibility coefficients (ADC, %) = [1 − (y*_i_*/y*_f_*) × (n*_f_*/n*_i_*)] (7)

where y*_i_* stands for the trioxide yttrium concentration in the feed, y*_f_* stands for the concentration in the feces, n*_i_* refers to the nutrient concentration in the feed, and n*_f_* refers to the concentration in the feces.

### 2.5. Nutritional Composition Analysis

The composition of the shrimp’s whole body and muscle was determined according to the national standards [22]. The moisture content of all samples was determined by drying at 105 °C in an oven until a constant weight was recorded. Ash analysis was performed by using combustion in a muffle furnace (FO610C, Yamato Scientific Co., Ltd., Tokyo, Japan) at 550 °C. Crude protein was quantified using an automatic Kjeldahl nitrogen analyzer (Gerhardt VAPODEST500, C. Gerhardt GmbH & Co.KG, Hamburg, Germany). Crude fat was quantified using a Soxhlet extraction system (Gerhardt Soxtherm, C. Gerhardt GmbH & Co.KG, Hamburg, Germany).

### 2.6. Antioxidant Enzyme Activity Analysis

The activity of superoxide dismutase (SOD), glutathione peroxidase (GPx), and the content of malondialdehyde (MDA), total protein (TP), as well as total antioxidant capacity (T-AOC), in the hepatopancreas were measured using the assay kits (Nanjing Jiancheng Bioengineering Institute, Nanjing, China). All supernatant samples were added to the reagents and incubated at 37 °C, the absorbance was measured with a microplate reader (Molecular Devices, CA, USA).

A unit of SOD activity is defined as the quantity of SOD needed to reduce the xanthine reduction rate by half in a 1 mL reaction mixture. Specific SOD activity is expressed as units per milligram of protein.

The activity of GSH-Px was measured by assessing the rate at which GSH is oxidized to glutathione (GSSG) due to H_2_O_2_. When GSH reacts with dithiobisnitrobenzoic acid, a yellow product that absorbs at 412 nm is produced [23]. One unit (U) of GSH-Px is characterized by its ability to decrease GSH concentration by 1 μmol L^−1^ in 5 min per milligram of hapatopancreas protein. The activity of GSH-Px in the hepatopancreas is measured in units per milligram of protein (U mg^−1^ protein).

MDA, which is byproduct of lipid peroxidation, can react with thiobarbituric acid (TBA) to produce a red compound that has its highest absorption at 532 nm. This technique is known as the TBA method due to the use of TBA as the substrate.

TP in the homogenate was measured using the Coomassie Brilliant Blue dye binding method, with bovine serum albumin serving as the standard [24]. The specific activity of enzyme is defined as activity unit per mg of protein.

### 2.7. Triglyceride and Fatty Acid Profile Analysis

TG and fatty acid composition in the hemolymph, liver and muscle were examined using Standard Testing Co., Ltd. (Qingdao, China). TG concentration is quantified using a sandwich immunoassay methodology. Microplate wells are coated with purified shrimp derived TG antibodies, establishing a solid-phase antibody layer. TG is then introduced to these coated wells, where it interacts with horseradish peroxidase (HRP) conjugated TG antibodies, resulting in the formation of an antibody–antigen-enzyme-labeled antibody complex. Subsequent to extensive washing, the substrate tetramethylbenzidine (TMB) is added to trigger chromogenic development. The HRP enzyme catalyzes TMB to produce a blue intermediate, which subsequently transitions to a yellow hue under acidic conditions. The intensity of this color change exhibits a direct correlation with the TG concentration in the specimen. Absorbance is measured at 450 nm using an enzyme-linked immunosorbent assay reader, and the TG concentration is determined by referencing a standard curve.

The fatty acid profile was performed using the method of GB 5009.168-2016 [25]. Fat Extraction Procedure: Accurately weigh an appropriate quantity of the sample into a 100 mL colorimetric tube. Add 8 mL of water and mix thoroughly. Subsequently, introduce 10 mL of hydrochloric acid and mix the solution again. Place the flask in a water bath maintained at 80 °C to facilitate hydrolysis for a duration of 1 h. During this period, agitate the flask every 30 min to ensure that any particles adhering to the flask walls are incorporated into the solution. Upon completion of the hydrolysis process, remove the sample and allow it to cool to room temperature. To the hydrolyzed sample, add 10 mL of 95% ethanol and mix thoroughly. Proceed with extraction in three portions using 100 mL of diethyl ether. Combine the extracts into a 100 mL flat-bottomed flask. Finally, evaporate the ether layer to dryness to obtain the extracted fat.

Saponification and fatty acid methylation of lipids were conducted as follows: To the lipid extract, 4 mL of a 2% sodium hydroxide solution in methanol was added. The mixture was incubated in a water bath maintained at 45 °C for 30 min. Subsequently, 4 mL of a 14% boron trifluoride solution in methanol was added, and the incubation at 45 °C was continued for an additional 30 min. Following the water bath treatment, the mixture was allowed to cool to room temperature. To facilitate extraction, 3 mL of n-Hexane was added to the centrifuge tube, and the mixture was shaken vigorously for 2 min. The mixture was then allowed to stand until phase separation occurred. The upper, clear layer was carefully transferred and filtered through a 0.45 μm membrane prior to instrumental analysis. The samples were analyzed using an Agilent 7890A gas chromatograph (Aglient Technologies, Palo Alto, CA, USA).

### 2.8. Lipid Metabolism-Related Enzyme Activity Analysis

The activity of total lipase (TL), hepatic lipase (HL), lipoprotein lipase (LPL), and lipase in the hepatopancreas was determined according to the instructions of assay kits (Nanjing Jiancheng Bioengineering Institute, Nanjing, China). The activity of fatty acid synthase (FAS), acetyl-CoA carboxylase (ACC) and adipose TG lipase (ATGL) carnitine palmitoyl transferase 1 (CPT-1) was quantified by assay kits based on enzyme-linked immunosorbent (Shanghai Enzyme-Linked Biotechnology Co., Ltd., Shanghai, China).

### 2.9. Antioxidant and Lipid Metabolism Gene Expression Analysis

All samples were thawed on ice after being taken from −80 °C. Then RNA was extracted using Trizol reagent based on instructions described by the supplier (Invitrogen, Shanghai, China). Synthesis of cDNA was performed according to the protocol of the Evo M-MLV reverse transcription premix kit (Accurate Biotechnology Code No. AG11728, Changsha, China). Real-time PCR amplification was conducted by SYBR Green 2^−∆∆ct^ relative quantification method [26] and the SYBR Green Pro Taq HS Premix qPCR Kit (Accurate Biotechnology Code No. AG11701, Changsha, China) according to the manufacturer’s instructions. After mixing the samples in PCR tubes, they were aliquoted into a 96-well PCR plate, briefly centrifuged, and subsequently placed into a PCR detection system (Heal Force CG-02, Shanghai, China) for amplification. Primers for real-time PCR were synthesized by Sangon Biotech (Shanghai, China). Table S1 presents all the primers used for real-time PCR amplification. The quantification results were analyzed using the following formulas:

∆ct = Ct value of candidate gene − Ct value of reference gene

∆∆ct = ∆ct of the experimental treatment − ∆ct of the control treatment.

### 2.10. Hepatopancreatic Lipidomics Analysis

The DL2, RL0.1, and RL2 groups were selected for lipidomic examination of hepatopancreatic samples. Lipid molecules were isolated following Mónica’s method [27] and analyzed using liquid chromatography-mass spectrometry [28]. Liquid chromatography was performed with an Agilent 1290 HPLC equipped with an ACQUITY UPLC^®^ BEH C18 1.7 µm (100 × 2.1 mm) column. Mass spectrometry was performed using an AB SCIEX AB6600 mass spectrometer with an electrospray ionization (ESI) source. Peak alignment, peak filtering, and lipid identification were performed using Lipid Search software (version 4.2.28) (Thermo Fisher Scientific, Waltham, MA, USA). Annotated lipids from the preprocessed data were classified based on fatty acyl chains and lipid classes. Multivariate statistical analysis was performed after data correction by using partial least squares discriminant analysis (PLS-DA) within the R package Ropls (version 1.22.0) (CeCILL, Grenoble, France). Differential lipid pathways were examined using the Biodeep cloud platform (https://www.biodeep.cn accessed on 10 June 2024).

### 2.11. Histological Morphological Observation

Tissue samples were paraffin-embedded and processed with high-definition constant staining pre-treatment solution, followed by staining with hematoxylin-eosin (H&E) and Oil Red O. Then samples were dehydrated and mounted using anhydrous ethanol and xylene. Digital scanning of the slides was performed using Pannoramic MIDI, Pannoramic 250 FLASH, and Pannoramic DESK (3DHISTECH, Budapest, Hungary). Taking images under white light illumination and subsequently scanning using digital slide CaseViewer software (version 2.4) (3DHISTECH, Budapest, Hungary).

### 2.12. Statistical Analysis

Data were presented as mean ± SD (standard deviation). Normality and homogeneity of variance were examined using Shapiro–Wilk and Levene model, respectively. One-way ANOVA (analysis of variance) was employed to examine the statistical difference of group means and Tukey’s test was chosen for post hoc multiple comparisons. Significant difference was considered with *p* < 0.05. In case parametric test was not applicable, a non-parametric Kruskal–Wallis test was used; For multiple comparison, Dunn’s test with Bonferroni adjustment was used to control Family-Wise Error Rate. All analysis was performed by using Statistical Package for the Social Sciences software (Version 27.0, SPSS, Chicago, IL, USA).

## 3. Results

### 3.1. Growth Performance of the Shrimp

The results are presented in Table 2. Shrimp in the RL0.1 group had the highest WG and SGR, while those in RL1.5 group showed the lowest FCR among all groups. LR increased progressively with rising lysolecithin addition, reaching significantly higher levels in RL1.5–RL2 groups compared to DL2 group (*p* < 0.05). These findings show lysolecithin contributed to lipid deposition in shrimp body.

The ADCs of protein and lipid of experimental diets for *L. vannamei* are shown in Table 3. The ADC of lipid demonstrated a biphasic response: values peaked in RL0.5 and RL1 groups (*p* < 0.05) but declined at higher concentrations (RL1.5–RL2.0 groups).

### 3.2. Nutritional Composition of the Whole Shrimp, Muscle, and Hepatopancreas

Progressive accumulation of crude fat in the whole shrimp, muscle, and hepatopancreas was observed with the increase in lysolecithin supplementation (Table 4). The crude fat in whole shrimp and muscle in the RL0.5-RL2 groups was significantly higher than that in the DL2 group (*p* < 0.05), while hepatopancreatic crude fat in the RL1, RL1.5, and RL2 groups were significantly higher than that in other groups (*p* < 0.05). Muscle protein declined substantially in RL2 (*p* < 0.05), possible due to the higher fat accumulation. These findings demonstrated that lysolecithin may increase systemic lipid accretion, with hepatopancreas showing notably responsive to 1–2% supplementation (*p* < 0.05).

### 3.3. Changes in the Antioxidant Function of the Hepatopancreas

#### 3.3.1. Antioxidant Enzyme Activity in the Hepatopancreas

The results are presented in Table 5. No significant differences of the tested antioxidant related parameters were observed in all groups, although hepatopancreatic total antioxidant capacity (T-AOC) and superoxide dismutase (SOD) were numerically decreased in the RL2 group compared to the DL2 group. Malondialdehyde (MDA) level in RL0.1 was the lowest in all groups.

#### 3.3.2. Relative Expression Levels of Antioxidant Genes in the Hepatopancreas

**Figure 1:**
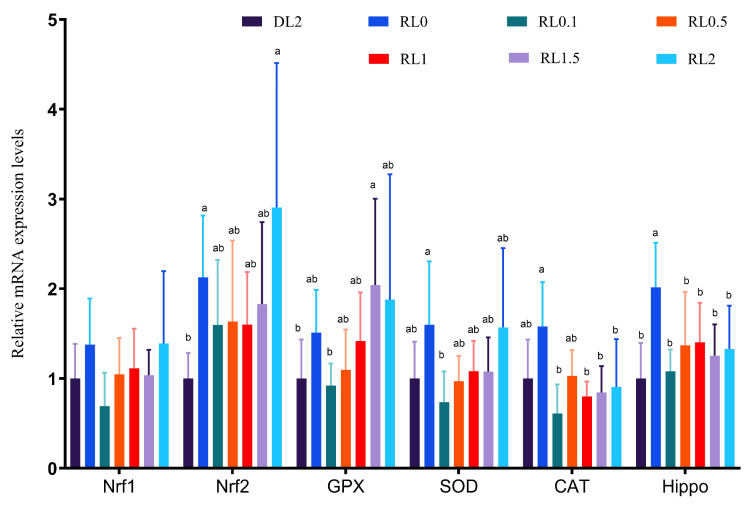
Effects of dietary lysolecithin on relative expression of antioxidant genes (*Nrf1*, *Nrf2*, *GPx*, *SOD*, *CAT*, *Hippo*) in the hepatopancreas of *L. vannamei*. Bars denote mean ± SD (*n* = 4). Different lowercase letters indicate significant differences (*p* < 0.05).

Figure 1 illustrates the relative expression levels of antioxidant genes in the hepatopancreas of *L. vannamei* in response to dietary lysolecithin. Expression levels of antioxidant genes generally increased with an increase in lysolecithin doses (0.1–2%). *Nrf1* and *CAT* expression in the RL0 group was significantly higher than that in the RL0.1 group (*p* < 0.05). Nuclear factor erythroid 2-related factors (*Nrf2*) and *SOD* expressions were significantly upregulated in RL0 and RL2 groups compared to the DL2 group (*p* < 0.05); *GPx* expression in RL1.5 and RL2 groups was significantly higher than that in the DL2 group (*p* < 0.05). The RL0 group exhibited significantly higher *Hippo* expression than all other groups (*p* < 0.05).

#### 3.3.3. Endoplasmic Reticulum (ER) Stress Genes in the Hepatopancreas

**Figure 2:**
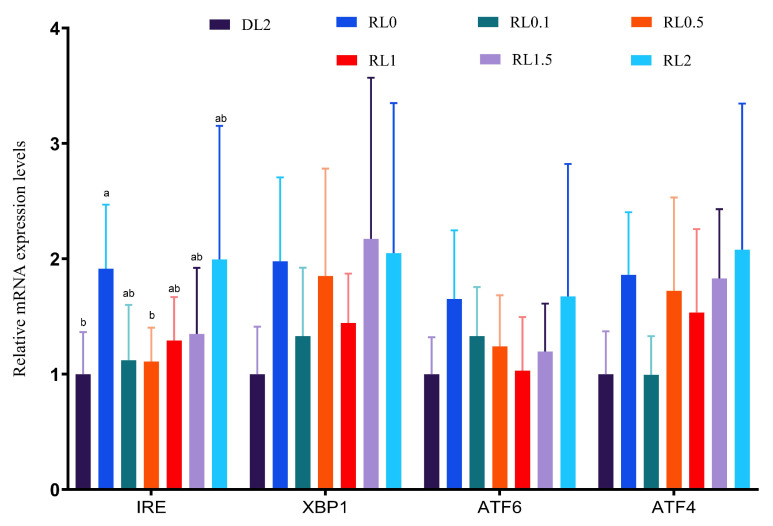
Effects of dietary supplemental lysolecithin on the expression of ER stress response genes (*IRE*, *XBP1*, *ATF6*, *ATF4*) in the hepatopancreas of *L. vannamei*. Bars denote mean ± SD (*n* = 4). Different lowercase letters indicate significant differences (*p* < 0.05).

The data is presented in Figure 2. ER stress-associated genes (*IRE*, *XBP1*, *ATF6*, and *ATF4)* were significantly induced in the RL0 and RL2 groups compared to the DL2 group (*p* < 0.05). However, *IRE* expressions in RL0.1, RL0.5, RL1, and RL1.5 groups significantly declined compared to RL0 group (*p* < 0.05). Compared to RL0 group, *ATF4* expression in RL0.1 group was significantly downregulated (*p* < 0.05). This implies that 0.1% lysolecithin bolster ER stress resilience, whereas deficiency (RL0) or excess (RL2) supplemental lysolecithin may impair adaptive capacity.

### 3.4. Changes in the Lipid Metabolism of the Shrimp

#### 3.4.1. Triglyceride Content

**Figure 3:**
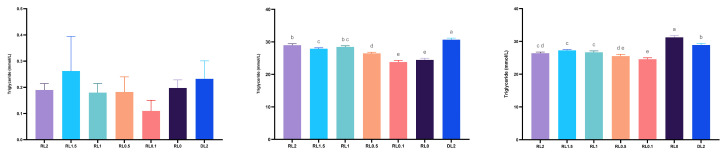
Effects of dietary treatments on TG levels in the hemolymph (**Left**), hepatopancreas (**Middle**), and muscle (**Right**) of shrimp *L. vannamei*. Bars indicate mean ± SD (*n* = 4). Different letters indicate significant differences between groups (*p* < 0.05).

Figure 3 illustrates the effects of dietary treatments on triglyceride (TG) content in the hemolymph, hepatopancreas, and muscle of *L. vannamei*. Hemolymph TG levels exhibited an initial increase followed by a decline as supplemental lysolecithin increased from 0.1% to 2%. Notably, TG content in the RL0.1 group was significantly lower than that in the DL2 group (*p* < 0.05). In the hepatopancreas and muscle, TG content increased progressively with higher levels of dietary lysolecithin, and the RL0.1 group had significantly lower values compared to other groups (*p* < 0.05). These findings indicate that supplemental 0.1% lysolecithin significantly reduced TG in the hemolymph, hepatopancreas, and muscle tissues of *L. vannamei*.

#### 3.4.2. Fatty Acid Profile in the Hepatopancreas and Muscle

The results demonstrated that varying levels of dietary lysolecithin supplementation caused distinct changes in the fatty acid profile in the hepatopancreas of *L. vannamei* (Table S2). Saturated fatty acids (SFAs) such as C14:0 and C15:0 initially decreased and then increased with higher lysolecithin levels, while others like C20:0, C21:0, and C23:0 exhibited the opposite trend. The highest SFA content was observed in the RL1 group, and the lowest in RL2, although these differences were not statistically significant compared to the DL2 control.

For monounsaturated fatty acids (MUFAs), C20:1 content increased and then decreased as lysolecithin increased, and the RL1 group shows a significantly higher C24:1 content than the other groups. However, no significant differences in MUFA were observed among all treatments.

Regarding polyunsaturated fatty acids (PUFAs), C18:3 content tended to increase with higher lysolecithin doses, while C20:3n3, C20:5n3, C22:6n3, and total n−3 PUFA content showed a decreasing trend. Notably, the RL0.1 group presented a significantly higher C22:6n3 and total n−3 PUFA content compared to the groups with higher lysolecithin. For n−6 PUFAs, C18:2n6c and C20:4n6 increased with lysolecithin, but some groups showed significantly lower levels than the control.

Dietary lysolecithin with various levels affected the fatty acid composition in the hepatopancreas; the most remarkable effects were observed at the dose of 0.1% lysolecithin, which increased specific n−3 PUFA content and the n−3/n−6 PUFA ratio. These findings indicate the importance of optimizing lysolecithin levels to improve the lipid quality of *L. vannamei*.

In the muscle tissue of *L. vannamei*, saturated fatty acid (SFA) contents did not differ significantly among groups (Table S3). Increasing dietary lysolecithin from 0.1% to 2% caused C16:1, C18:1n9, C20:1, and total MUFA content to rise and then fall. The RL0.5 group showed notably higher C16:1 and C18:1n9 than the DL2 group (*p* < 0.05).

The ∑n−3/∑n−6 PUFA ratio decreased as dietary lysolecithin levels increased, but remained significantly higher in RL0.1 and RL0.5 compared to the DL2 group (*p* < 0.05). Dietary lysolecithin changed muscle fatty acid profiles in *L. vannamei*, especially MUFA, PUFA, and the ∑n−3/∑n−6 ratio, with significant changes observed at dosages of 0.1–0.5%.

#### 3.4.3. Lipid Metabolism Enzymes in the Hepatopancreas

Increasing dietary lysolecithin from 0.1% to 2% increased the activities of TL, HL, and LPL enzymes in the hepatopancreas, and RL1.5 and RL2 groups exhibited significantly higher activities than the DL2 group (*p* < 0.05). The activities of ACC, FAS, CPT-1, and ATGL tended to increase with the increase in lysolecithin dosages. ACC and FAS activities in the RL1.5 group are significantly higher than those in the DL2 group (*p* < 0.05). The results suggest that dietary lysolecithin at dosages of 1% to 2% significantly enhanced lipid metabolic enzyme activities in the hepatopancreas of *L. vannamei* (Table 6).

#### 3.4.4. Expression of Lipid Metabolism Genes in the Hepatopancreas

**Figure 4:**
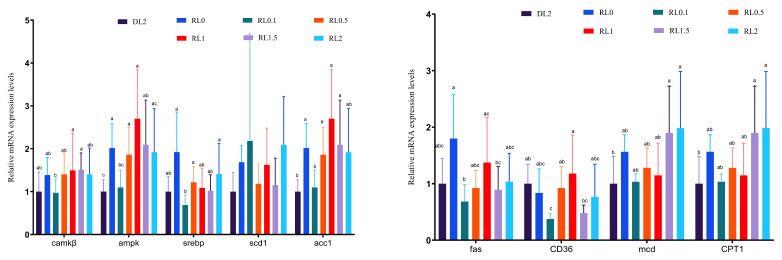
Gene expressions involved in hepatopancreatic lipid metabolism (*camkkβ*, *ampk*, *srepk*, *acc1*, *fas*, *scd1*, *cd36*, *mcd*, *cpt1*) in *L. vannamei* fed with different diets. Bars represent the mean ± SD (*n* = 4). Different letters indicate significant differences between groups (*p* < 0.05).

Figure 4 depicts the relative expression of hepatopancreatic lipid metabolism genes in *L. vannamei*. As the lysolecithin level in the feed increased from 0.1% to 2%, hepatopancreatic acc1, scd1, mcd, and cpt1 relative expressions tended to increase gradually, and *camkkβ*, *ampk*, *srebp*, and *cd36* relative expression tended to increase initially and subsequently decrease. The relative expression of *camkkβ* in the RL1 and RL1.5 groups was significantly higher than that in the DL2 group (*p* < 0.05). The relative expressions of *camkkβ* in RL0, RL0.5, RL1, RL1.5, and RL2 groups were significantly higher than those in the DL2 group (*p* < 0.05). The relative expressions of *SREBP* in the RL0 and RL1 groups were significantly higher than those in the DL2 group (*p* < 0.05), and the relative expression of *ACC1* in the RL0 and RL2 groups was significantly higher than that of the DL2 group (*p* < 0.05). The relative expression of *Fas* in the RL0.1 and RL2 groups was significantly higher than that in the DL2 group (*p* < 0.05). The relative expressions of SCD1 in the RL0.5 and RL2 groups were significantly higher than those in the DL2 group (*p* < 0.05). The relative expressions of *mcd* and *cpt1* in RL0, RL1.5, and RL2 groups were significantly higher than those in the DL2 group (*p* < 0.05), and the relative expression of *cd36* in RL0.1 and RL1.5 groups was significantly lower than that in the DL2 group (*p* < 0.05). The results demonstrated that the addition of 1–2% lysolecithin could significantly increase the relative expression levels of hepatopancreatic lipid metabolism genes in *L. vannamei*. The expression levels of genes involved in hepatopancreatic lipid metabolism in *L. vannamei* were examined at different dietary lysolecithin dosages. Increasing lysolecithin from 0.1% to 2% upregulated *acc1*, *scd1*, *mcd*, *and cpt1* mRNA levels overall, while *camkkβ*, *ampk*, *srebp*, *and cd36* increased first, then decreased. *Camkkβ* expression in the RL1 and RL1.5 groups was higher than that in the DL2 group (*p* < 0.05). Additionally, the expression of *camkkβ* in the RL0, RL0.5, RL1, RL1.5, and RL2 groups was also upregulated compared to the DL2 group (*p* < 0.05). *SREBP* expressions in RL0 and RL1 were significantly increased compared to DL2 (*p* < 0.05), and *ACC1* expressions in RL0 and RL2 were also significantly higher (*p* < 0.05). *Fas* expression was significantly greater in RL0.1 and RL2 than in DL2 (*p* < 0.05). *SCD1* expression levels in the RL0.5 and RL2 groups were significantly higher than those observed in the DL2 group (*p* < 0.05). The expressions of *mcd* and *cpt1* were significantly increased in RL0, RL1.5, and RL2 groups compared to DL2 (*p* < 0.05), whereas *cd36* expression was significantly lower in RL0.1 and RL1.5 groups relative to DL2 (*p* < 0.05). These findings demonstrate that dietary lysolecithin at 1–2% significantly enhanced the expression of hepatopancreatic lipid metabolism genes in *L. vannamei*.

#### 3.4.5. Hepatopancreas Lipidomics Changes

##### Lipid Composition

The hepatopancreatic lipid of *L. vannamei* in DL2, RL0.1, and RL2 groups was analyzed using liquid chromatography–tandem mass spectrometry. 1768 lipids were identified in the hepatopancreas of *L. vannamei*, which were classified into 52 categories. Except for phosphatidylcholine (PC) in the RL0.1 group at 20.95%, all other lipid types were consistent (Figure S1). The main lipid categories were PC (20.9%), TG (20.9%), phosphatidylethanolamine (PE) (8.14%), diglyceride (DG) (11.30%), and sphingomyelin (SM) (6.87%).

##### Lipid Identification and Verification

**Figure 5:**
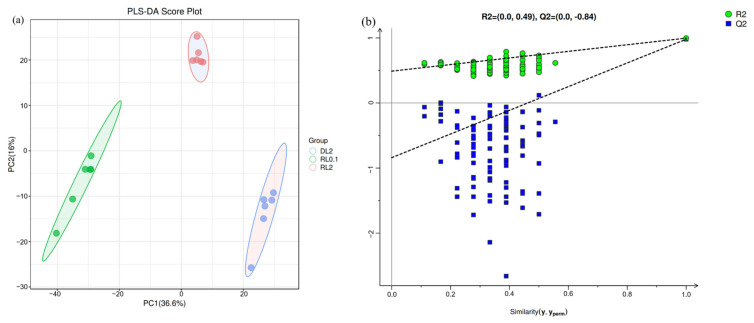
PLS-DA analysis of the hepatopancreas lipidome of *L. vannamei*. (**a**) PLS-DA score plot. (**b**) Permutation test plot.

PLS-DA is a classification technique widely used in metabolomics data analysis. The analysis showed that the three groups, DL2, RL0.1, and RL2, were completely separate, suggesting statistically significant differences between the groups (*p* < 0.05) (Figure 5a). The results indicate that dietary treatments significantly altered the lipid profiles of the hepatopancreas in *L. vannamei*. R2X, R2Y, and Q2 are key metrics in PLS-DA: R2X measures explanatory power for X variables, R2Y for Y variables, and Q2 indicates predictive strength. In Figure 5b, all Q2 points fall below the furthest original Q2 (aligned with upper right R2), and the regression line crosses the vertical axis below zero, supporting the results’ reliability.

##### Differential Lipid Analysis

**Figure 6:**
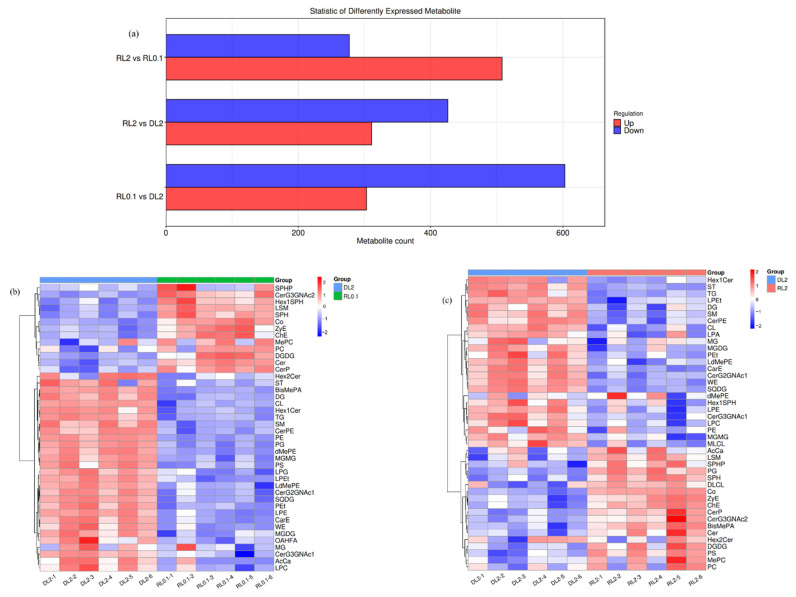
(**a**) Heatmap of the lipid subclasses abundance in the hepatopancreas of *L. vannamei*. (**b**) Comparison between the RL0.1 and DL2 groups. (**c**) Comparison between the RL2 and DL2 groups.

The screening criteria for differential lipids were set as *p* < 0.05 and fold change ≥ 1.5 or ≤0.67. Compared to the DL2 group, the RL0.1 group exhibited 303 upregulated and 603 downregulated lipids, whereas the RL2 group exhibited 311 upregulated and 426 downregulated lipids. Compared to the RL0.1 group, the RL2 group exhibited 508 upregulated and 277 downregulated lipids. The significant differences in major lipid subclasses were statistically analyzed (Figure 6, Table S4). The results revealed that compared to the DL2 group, 31 and 16 differential lipids were identified in the RL0.1 and RL2 groups, respectively. Among phospholipids, cardiolipin (CL, DLCL, and MLCL), lysophosphatidic acid (LPA), phosphatidylinositol (PI), and lysophosphatidylserine (LPS) were elevated in the RL0.1 group, while lyso-phosphatidylethanolamine (LPE), phosphatidylethanolamine (PE), and lyso-phosphatidylglycerol (LPG) were decreased, whereas phosphatidic acid (PA) and phosphatidylglycerol (PG) were elevated, and LPA was decreased in the DL2 group.

Among sphingolipids, ganglioside (GM3), lysosphingomyelin (LSM), and sphingo myelin (SPHP) were elevated in RL0.1 and DL2 groups, while ceramide phosphoethanolamine (CerPE) decreased. Hexosylceramide (Hex1SPH) and sphinganine (SPH) exhibited increased levels in the RL0.1 group. In the simple ganglioside series, CerG3GNAc2 was increased and CerG2GNAc1 was decreased in the RL0.1 and RL2 groups, whereas Hex1Cer and CerG3GNAc1 decreased in the RL0.1 group. Among neutral lipids, cholesterol ester (ChE), TG, and zymosterol ester (ZyE) were elevated in the RL0.1 group. In fatty acyls and other lipids, coenzyme (Co) was elevated, and lysophosphatidylethanol (LPEt) was decreased in the RL0.1 and DL2 groups.

Among glyceroglycolipids, monogalactosyl monoacylglycerol (MGMG), monogalactosyl diacylglycerol (MGDG), and sulfoglycolipid diacylglycerol (SQDG) were decreased in the RL0.1 and DL2 groups, while digalactosyl diacylglycerol (DGDG) was elevated in the RL0.1 group. Among lipid derivatives, phosphoethanolamine-N-(biotin) (BiotinylPE) was elevated in the RL0.1 and DL2 groups, while dimethyl phosphatidic acid (BisMePA) was decreased in the RL0.1 group. Among acylcarnitines, carnitine ester (CarE) was decreased in the RL0.1 and DL2 groups. The differential lipid screening criteria were set as *p* < 0.05 and fold change ≥1.5 or ≤0.67. Compared to the DL2 group, the RL0.1 group exhibited 303 upregulated and 603 downregulated lipids, while the RL2 group showed 311 upregulated and 426 downregulated lipids. In comparison between the RL2 and RL0.1 groups, 508 lipids were upregulated and 277 downregulated. Statistical analysis of major lipid subclasses revealed that, relative to the DL2 group, 31 differential lipids were identified in the RL0.1 group and 16 in the RL2 group. Among phospholipids, cardiolipin (CL, DLCL, MLCL), lysophosphatidic acid (LPA), phosphatidylinositol (PI), and lyso-phosphatidylethanolamine (LPE), phosphatidylethanolamine (PE), and lyso-phosphatidylglycerol (LPG) decreased. In contrast, phosphatidic acid (PA) and phosphatidylglycerol (PG) increased, and LPA decreased in the DL2 group. For sphingolipids, ganglioside (GM3), lyso-sphingomyelin (LSM), and sphingomyelin phosphate (SPHP) were elevated in RL0.1 and DL2 groups, but ceramide phosphoethanolamine (CerPE) decreased. Hexosylceramide (Hex1SPH) and sphinganine (SPH) increased in RL0.1. Within simple gangliosides, CerG3GNAc2 was increased, while CerG2GNAc1 decreased in RL0.1 and RL2 groups, and Hex1Cer and CerG3GNAc1 decreased in RL0.1. Regarding neutral lipids, cholesterol ester (ChE), triglyceride (TG), and zymosterol ester (ZyE) were elevated in RL0.1. In the fatty acyl and other lipid categories, coenzyme (Co) increased, and lyso-phosphatidylethanol (LPE) decreased in RL0.1 and DL2. Among glyceroglycolipids, monogalactosyl monoacylglycerol (MGMG), monogalactosyl diacylglycerol (MGDG), and sulfoglycolipid diacylglycerol (SQDG) decreased in RL0.1 and DL2 groups, while digalactosyl diacylglycerol (DGDG) increased in RL0.1. Regarding lipid derivatives, phosphoethanolamine-N-(biotin) (BiotinylPE) increased in RL0.1 and DL2, whereas dimethyl phosphatidic acid (BisMePA) decreased in RL0.1. For acylcarnitine, carnitine ester (CarE) decreased in RL0.1 and DL2. Different dietary treatments induced significant changes in the hepatopancreatic lipid profiles of *L. vannamei*, primarily reflected in distinct shifts among phospholipids, sphingolipids, neutral lipids, glyceroglycolipids, lipid derivatives, and acylcarnitine. These results indicate that dietary lysolecithin may play a pivotal role in modulating lipid metabolism in shrimp hepatopancreas.

##### Analysis of Lipid Structural Characteristics and Functional Enrichment

**Figure 7:**
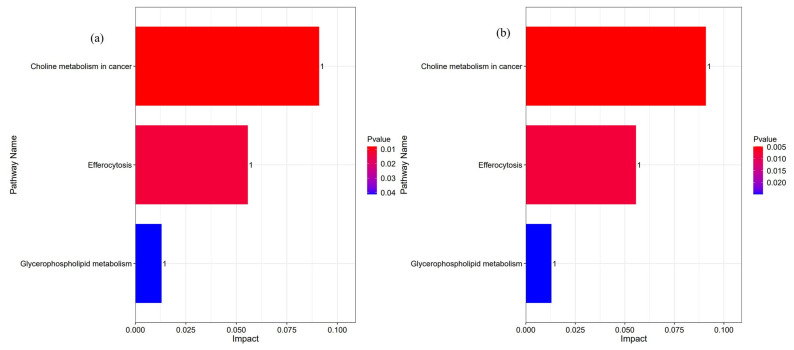
KEGG pathways enriched for differential lipids in the hepatopancreas of *L. vannamei*. (**a**) A Comparison of RL0.1 with DL2 groups. (**b**) A comparison of the RL2 with the DL2 groups.

Additional analysis was focused on the pathway involved in all differentially expressed lipids. Three identical pathways were observed in the RL0.1 and RL2 groups, compared to the DL2 group, primarily linked to “glycerophospholipid metabolism,” “phagocytosis,” and “choline metabolism” (Figure 7).

### 3.5. Histological Changes of the Hepatopancreas

#### 3.5.1. HE-Stained Sections of the Hepatopancreas

**Figure 8:**
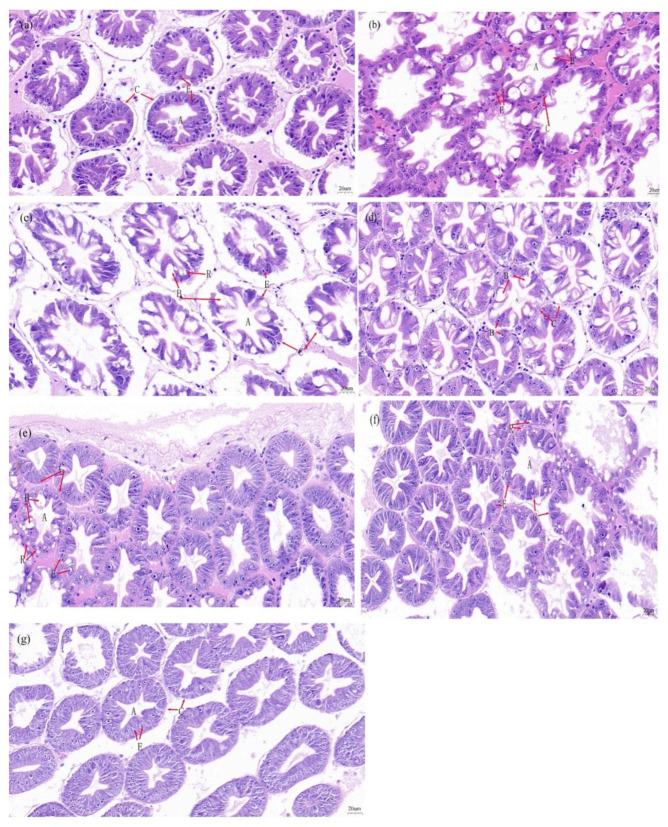
HE—staining (×400) hepatopancreas sections of *L. vannamei* fed with different diets. (**a**) DL2 group; (**b**) RL0 group; (**c**) RL0.1 group; (**d**) RL0.5 group; (**e**) RL1 group; (**f**) RL1.5 group; (**g**) RL2. Group. In the figure, A denotes the astrocytic lumen, C denotes the basement membrane, B denotes the B cell (secretory), R denotes the R cell (storage), and E denotes the E cell (embryonic).

Figure 8 presents the HE stained sections of the hepatopancreas of *L. vannamei*. In the DL2 group, the hepatic tubules were scattered, predominantly comprising E-cells, with fewer B-cells and R-cells, and the basement membranes were visible. The RL0 group showed an increased number of B-cells, a deformed stellate cavity, partial basement membrane was damaged, and large cell size, which made R-cells less discernible. For RL0.1 group, the hepatic tubules were relatively concentrated, and B-cells and R-cells were more concentrated, and the basement membrane was visible. In the RL0.5 group, the hepatic tubules arrangement was fuller and tighter, with more B and R cells, and the basement membrane was visible. In the RL1 group, the hepatic tubules were tightly arranged, and the number of E cells began to increase. In the RL1.5 group, the hepatic tubules arrangement was looser, with more R cells and E cells, and some of the stellate cavity began to deform. In the RL2 group, the hepatic tubules were more dispersed, with more E cells and fewer B and R cells.

#### 3.5.2. Oil Red O Sections of the Hepatopancreas

**Figure 9:**
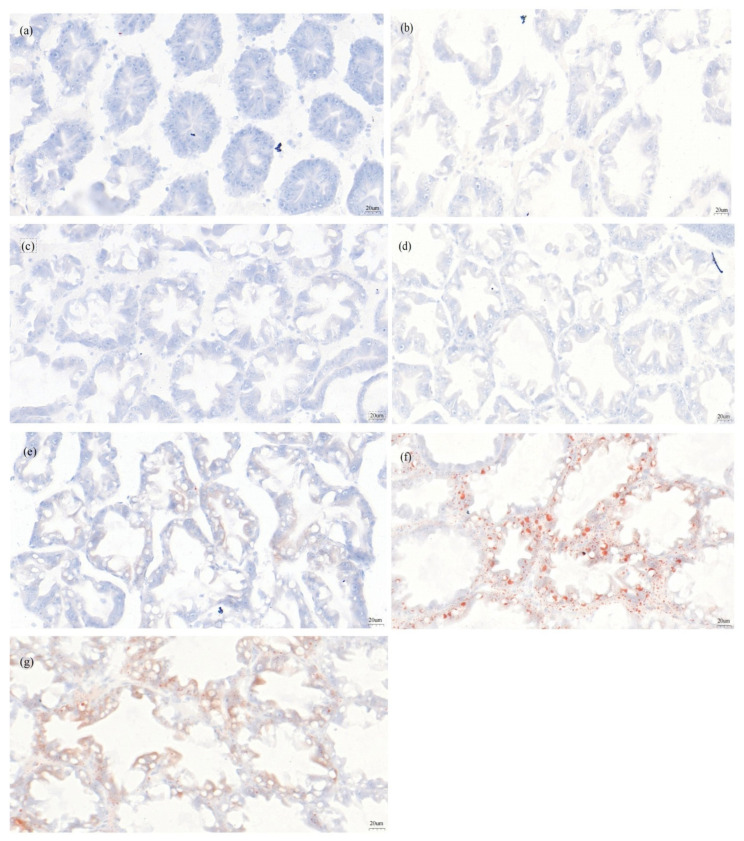
Oil red O-stained hepatopancreas sections (×400) of *L. vannamei*: (**a**) DL2 group; (**b**) RL0 group; (**c**) RL0.1 group; (**d**) RL0.5 group; (**e**) RL1 group; (**f**) RL1.5 group; (**g**) RL2 group.

Figure 9 shows oil red O staining of the shrimp hepatopancreas across dietary treatments. As lysolecithin levels increased, lipid droplets and the oil red O staining became more prominent. The RL1~2.0 groups exhibited significantly more fat vacuoles and dispersed cells than DL2. These results indicate that lysolecithin supplementation notably promotes lipid droplet formation in the hepatopancreas, which consists with the results obtained in hepatopancreatic fat content. The qualitative analysis provides preliminary descriptive support.

## 4. Discussion

### 4.1. Effects of Dietary Growth Performance of the Shrimp

Feed digestibility is affected by the chemical composition, the farming species, as well as the digestive traits [29]. Deng et al. [30] conducted a study on soy-based emulsifiers enriched with lyso-phosphatidylcholine, highlighting its capacity to stabilize food emulsions through adsorption at the oil-water interface. Law et al. [31] examined the wider metabolic pathways of lyso-phosphatidylcholine. Its role in biological processes and various applications was comprehensively described, including its function as an emulsifier in different systems. Researchers proposed that the emulsifying property of lyso-phosphatidylcholine (LPC) could improve the surface activity of lipids and convert them into micelles, improving nutrient utilization. Zhao et al. [32] conducted an experiment that utilized lyso-phosphatidylcholine to enhance the apparent digestibility of crude fat in weaned piglets. This study demonstrated that an increase in lysolecithin levels in the feed corresponded with a gradual stabilization of the LR of *L. vannamei*. The supplementation of lysolecithin resulted in significantly higher digestibility of crude fat compared to the soy lecithin control group, suggesting that optimal dietary lysolecithin could improve fat utilization and obtain better growth of *L. vannamei* when substitute to 2% of soy lecithin in shrimp diets.

### 4.2. Effects of Dietary Lysolecithins on Nutritional Composition of Shrimp

Lysolecithins are glycerophospholipids removed one fatty acid chain, with only a single hydroxyl group on the glycerol backbone [33]. They trigger cell signaling and have various physiological functions in humans and other animals. This study demonstrated that crude fat content in whole shrimp, muscle, and hepatopancreas of *L. vannamei* increased significantly with increasing levels of lysolecithin added to the diet, with crude fat significantly higher than that of the control group at 0.5–2% levels of lysolecithin supplemented. However, Liu et al. [11] reported that dietary lysolecithin levels (0.0125–0.05%) reduced whole fish and liver lipid content in channel catfish (*Ictalurus punctatus*). Xu et al. [34] reported that dietary lysolecithin levels (0.1–0.5%) exhibited no significant effect on whole body fat in young chickens. Li et al. [10] stated that dietary lysolcithin levels (0.1–0.55%) significantly increased the crude fat content of juvenile turbot (*Scophthalmus maximus*) livers, and these findings are consistent with those of this study. The difference in these results may be due to the emulsifier types and dosages.

### 4.3. Effects of Dietary Lysolecithins on Antioxidant Capacity of Shrimp

Oxidative stress, characterized by an imbalance between the generation of reactive oxygen species (ROS) and the organism’s antioxidant defense mechanisms, represents a fundamental biological challenge encountered by nearly all aerobic organisms, ranging from microorganisms to humans [35]. The principal antioxidant enzyme system, comprising SOD, CAT, and GPx, serves as a highly conserved primary defense mechanism to neutralize ROS and mitigate cellular damage [36]. This system is predominantly regulated at the transcriptional level by the Nrf2-Keap1 signaling pathway, which is activated in response to oxidative stress [37]. T-AOC quantifies the overall capacity of the antioxidant system in an organism [38]. The antioxidant enzyme system removes ROS, helping to keep the intracellular environment balanced and supporting normal cellular function. Therefore, the antioxidant enzyme system is essential for preserving the health of organisms and resisting oxidative stress [39]. The hepatopancreas is the center of metabolic processes in shrimp, where antioxidant enzymes contribute to protection against oxidative stress and are crucial to energy metabolism and immunological defense [40]. The results of this study show that, compared to the control group DL2, there were no significant differences in the expression levels of genes such as SOD, GPx, CAT or in the corresponding enzyme activities among the other groups except for RL0. This indicates that, under the conditions of this experiment, dietary lysolecithin supplementation has a limited effect on the conserved antioxidant system in the hepatopancreas of *L. vannamei*. MDA, a byproduct of lipid peroxidation, is extensively utilized as a biomarker for evaluating oxidative stress levels [41]. In the RL0.1 group, its presence suggests a reduced degree of oxidative damage to cellular membranes. Previous studies indicated that dietary lysolecithin enhanced antioxidant capacity and mitigate inflammation in both fish and pigs [12,42,43]. Our findings differ from those reported in some other species, suggesting that the antioxidant effects of lysolecithin may be influenced by factors such as species, dosage, and feed formulation. Therefore, the antioxidant mechanisms and effects of lysolecithin in *L. vannamei* require further investigation. It should be noted that gene expression levels do not always align with protein function, and factors such as post-transcriptional modifications or enzyme inhibition may explain differences observed.

Nrf2 regulates the expression of various antioxidant enzymes, such as GPx, CAT, and SOD, by binding to antioxidant response elements (AREs). These genes act as antioxidants and help protect cells [44]. The *Hippo* pathway is crucial for regulating cell proliferation and apoptosis and participates in antioxidant responses. The *Hippo* pathway regulates the downstream *YAP/TAZ* transcription factors, which influence the gene expression of antioxidant enzymes and increase the resistance to environmental stress [45]. *Nrf2* interacts with the *Hippo* pathway to co-regulate cellular antioxidant systems. It influences antioxidant enzyme expression indirectly by modulating *YAP/TAZ* activities [46]. This study found that the gene expression of *Nrf1*, *Nrf2*, *GPx*, *SOD*, *CAT*, and *Hippo* in the hepatopancreas gradually increased as lysolecithin dosage increased from 0.1% to 2%. These results suggest that lysolecithin may help maintain the health of *L. vannamei* by enhancing its free radical scavenging activity and reducing oxidative stress [47]. However, the antioxidant function of lysolecithin is not well understood, and no significant differences in antioxidant gene expression were observed in hepatopancreas compared to the control group. This may be related to β-oxidation gene activity, and the link between β-oxidation and antioxidant responses requires further investigation [48].

ER stress studies in shrimp have demonstrated its important role in response to environmental and pathogenic stress [49]. ER stress regulates cellular self-defense mechanisms through the activation of the unfolded protein response (UPR). The UPR is primarily regulated through three signaling pathways: the PERK, the ATF6, and the IRE1. Specifically, the PERK pathway regulates cellular self-defense by phosphorylating EIF2α, thereby inhibiting protein synthesis and alleviating ER burden; the ATF6 pathway activates ER-associated gene expression by cleaving and translocating them to the nucleus; and the IRE1 pathway regulates protein folding and degradation by generating functional XBP1 protein through shearing XBP1 mRNA [50]. Our data suggests that lysolecithin supplementation mitigates endoplasmic reticulum (ER) stress in shrimp, as demonstrated by the downregulation of key UPR markers, including *IRE1*, *XBP1*, *ATF6*, and *ATF4*. ER stress represents a highly conserved cellular response to the accumulation of misfolded proteins and is intricately associated with lipid metabolism and oxidative stress. This finding is consistent with research conducted in mammalian liver cells, where lipid overload-induced ER stress was alleviated by various bioactive lipids or emulsifiers [51]. Furthermore, a study conducted on yellow-feathered broilers demonstrated that dietary supplementation with a different emulsifier reduced ER stress and enhanced lipid homeostasis [52]. The conserved nature of the UPR across different taxa underscores the biological significance of our observations in shrimp. It is plausible that by enhancing lipid emulsification and absorption, lysolecithin reduces the metabolic burden on the hepatopancreas, thereby preventing the lipotoxicity that often triggers ER stress. A less stressed ER environment would, in turn, contribute to improved cellular homeostasis and overall health, which aligns with our histological observations.

In summary, this study extends the application of dietary lysolecithin beyond shrimp aquaculture by demonstrating its role as a significant modulator of the highly conserved pathways involved in oxidative stress and cellular homeostasis. The findings indicate that lysolecithin enhances the Nrf2-mediated antioxidant response and alleviates endoplasmic reticulum stress, suggesting its potential as a broad strategy to enhance stress resilience and metabolic health across a diverse array of organisms facing oxidative challenges.

### 4.4. Effects of Dietary Lysolecithins on the Lipid Metabolism of Shrimp

Lysolecithin can affect lipid accumulation. TG is an important product and intermediate in lipid metabolism in vivo, with high levels of TGs usually associated with diseases, including metabolic syndrome and nonalcoholic fatty liver disease [53]. This study examined the biochemical index TG concentrations in the tissues of *L. vannamei* to understand its lipid metabolism. The experimental results revealed that supplemental 0.1% lysolecithin to the diet significantly diminished the hemolymph, hepatopancreas, and muscle TG content in *L. vannamei*, and it was initially inferred that lysolecithin could reduce lipid accumulation by modulating lipid metabolism and signaling pathways. The addition of β-sitosterol significantly reduces TG and cholesterol accumulation in zebrafish in a study using a high-fat diet-induced model of nonalcoholic fatty liver disease [54]. Regarding fatty acid composition, as the level of lysolecithin in the feed increased from 0.1% to 2%, the shrimp hepatopancreas ∑n−3 PUFA content was significantly reduced, ∑n−6 PUFA content was significantly increased, ∑n−3/∑n−6 PUFA content was significantly reduced, and ∑n−3/∑n−6 PUFA was significantly increased with addition of 0.1% lysolecithin compared to the control group. Similar results were found in shrimp muscle. A higher ∑n−3/∑n−6PUFA ratio usually indicates that the body is enriched with more omega-3 fatty acids, including eicosapentaenoic acid (EPA) and docosahexaenoic acid (DHA), which greatly benefit shrimp health [55].

Further studies have demonstrated that lysolecithin facilitates lipid transport and metabolism by enhancing the emulsification and absorption of fats, which subsequently affects the activity of enzymes associated with lipid metabolism. Hepatopancreas, as the central organ of lipid metabolism in shrimp, regulates fatty acid synthesis and catabolism, hence maintaining lipid homeostasis in the body. Its functionality is mediated by the activities of enzymes, including TL, HL, LPL, Lipase, ACC, FAS, CPT-1, and ATGL [15]. TL includes all types of lipases in the body that are involved in lipid catabolism. TL is a crucial process in lipid metabolism, facilitating the hydrolysis of TGs and the release of fatty acids and glycerol. HL mediates the hydrolysis of circulating TGs and phospholipids, regulating plasma high-density lipoprotein and low-density lipoprotein in plasma, thereby influencing cholesterol transport and metabolism, which is significant in atherosclerosis development. LPL is responsible for the hydrolysis of TGs in plasmachyme and very low-density lipoproteins, which play a vital role in lipid transport from the bloodstream to tissues, thus regulating energy supply and fat storage [56]. Herein, we demonstrated that TL, HL, and LPL activities were significantly increased with the addition of higher levels of lysolecithin to the feed, and the addition of lysolecithin increased TL, HL, and LPL activities compared to the control group. ACC is essential in fatty acid synthesis, converting acetyl coenzyme A to malonyl coenzyme A, which is the first step in long-chain fatty acid biosynthesis; FAS is a pivotal lipid biosynthesis enzyme that transforms malonyl coenzyme A to palmitic acid. CPT-1 facilitates the β-oxidation of fatty acids, which helps transport the long-chain fatty acids into the mitochondria to produce energy. ATGL serves as the rate-limiting enzyme in lipolysis, hydrolyzing TGs into free fatty acids and glycerol [57]. This study demonstrated that ACC and FAS activities were significantly elevated at a 1.5% level of lysolecithin addition compared to the control group, while CPT-1 and ATGL activities were elevated with the addition of lysolecithin. These findings indicate that lysolecithin can modulate lipid metabolic pathways by modifying enzyme activity and substrate accessibility. Understanding these mechanisms may facilitate the formulation of new dietary supplementation techniques to enhance lipid metabolism and prevent associated diseases.

An expression analysis of relevant genes was performed to elucidate the molecular regulatory mechanisms involved in the impact of lysolecithin on hepatic lipid metabolism. Within the Ca^2+^/CaMKKβ/AMPK pathway, calcium ions bind to calmodulin, forming the Ca^2+^/CaM complex. This complex subsequently associates with calmodulin-dependent protein kinase kinase β (CaMKKβ), inducing a conformational change that activates CaMKKβ. The activated CaMKKβ then phosphorylates AMPK at the Thr172 residue within its activation loop, thereby initiating AMPK activity [58]. AMPK activation modulates cellular energy metabolism by enhancing glucose uptake, facilitating fatty acid oxidation, and stimulating mitochondrial biosynthesis, while also accelerating fatty acid catabolism and inhibiting fatty acid and cholesterol synthesis [59]. AMPK can diminish the activity of sterol regulatory element-binding protein-1c (SREBP-1c) through direct or indirect mechanisms [60]. SREBP-1c acts as a transcription factor, enhancing the gene expression related to fatty acid synthesis. AMPK activation inhibits the maturation and activity of SREBP-1c, which reduces the expression of fatty acid synthetases, including acetyl-coenzyme A carboxylase (ACC), fatty acid synthetase (FAS), and stearoyl CoA desaturase (SCD1), and other lipid synthetases [58]. AMPK is implicated in lipolysis metabolism. When AMPK is activated, CD36 is translocated to the cell membrane, enhancing fatty acid uptake. Samovski et al. [61] reported that AMPK activation results in increased CD36 expression and activity at the cell surface, thereby combining fatty acid uptake with fatty acid catabolism. AMPK enhances fatty acid absorption by regulating fatty acid oxidation-related genes in the oxidative utilization in mitochondria. These genes include carnitine palmitoyl transferase (CPT-1), a crucial enzyme facilitating fatty acid entry into the mitochondria for β-oxidation [62]. Shi et al. [63] reported that AMPK activation enhanced MCD activity and reduced the level of malonyl coenzyme A, an inhibitor of CPT-1, indirectly facilitating the transport and subsequent oxidation of fatty acids and thus increasing the ATP production. This study demonstrated that the addition of lysolecithin at 1%, 1.5% and 2% levels into the feed significantly enhanced the gene expression of *camkkβ*, *ampk*, *cd36*, *mcd*, and *cpt1* in the hepatopancreas of *L. vannamei*. The simultaneous upregulation of CaMKKβ/AMPK, along with alteration in downstream lipid metabolism gene, indicates that Ca^2+^/CaMKKβ/AMPK signaling pathway may be activated by dietary lysolecithin. This results is consistent with the findings of Shi et al. [63]. Compared with the group lacking lysolecithin, the expression of *SREBP*, *ACC1*, *FAS*, AND *SCD1* genes was reduced by the addition of lysolecithin, and the results were more significant at the level of 0.1%. Lysolecithin maybe inhibits the binding of the transcription factor SREBP to the downstream *FAS*, *ACC1*, and *SCD1*, leading to a decrease in the expression of these genes and reducing lipid synthesis and deposition. However, the exact mechanism through which dietary lysolecithin affects lipid metabolism requires additional research. Future investigations should utilize Western blot analysis to assess the phosphorylation status of AMPK at Thr172 and its direct targets, such as ACC, to verify pathway activation. Additionally, conducting in vitro or in vivo pathway inhibition experiments, for instance, using compound C or specific siRNA, will be essential to establish a definitive causal link between AMPK activation and the observed metabolic enhancements.

Combined with the results of hepatopancreatic HE-staining sections, the control group exhibited a dispersed arrangement of liver vesicles, predominantly comprising E cells, with fewer B and R cells; in the group without lysolecithin, the central stellate lumen appeared deformed, portions of the basement membrane were compromised, and the cell volume was enlarged. Conversely, after the addition of lysolecithin, the arrangement of liver vesicles was fuller and more compact, with more B and R cells. The basement membrane was visible, and the cellular morphology appeared more uniform and orderly. After the addition of lysolecithin, liver microsomes were arranged more fully and tightly, with more Band R cells. The basement membrane was visible, and the cellular morphology appeared more uniform and orderly. The results of oil red O-stained sections of hepatopancreas demonstrated that the hepatic microsomes were more structurally complete and tightly arranged after the addition of lysolecithin. After adding lysolecithin to the hepatopancreas, the level gradually increased to 2%. There was no abnormal fat accumulation in the hepatopancreas, nor were there fat vacuoles in the R cells. This further substantiates that the addition of lysolecithin safeguards the hepatopancreatic hepatic microsomal structure, enhances lipid absorption and metabolism in shrimp, and aids in preserving the normal functionality of the hepatic microsomes. Additionally, the high dosage of lysolecithin does not adversely influence the hepatopancreatic lipid metabolism of shrimp. Chen et al. [64] reached similar conclusions on the effects of different fatty acid sources and emulsifier additions on the hepatopancreas of *L. vannamei*. Whether the addition of lysolecithin is beneficial in reducing the resistance of shrimp to environmental stress or disease remains to be verified.

### 4.5. Effects of Dietary Lysolecithins on the Hepatopancreas Lipidomics of Shrimp

Hepatopancreatic lipidomics analysis further confirmed that the addition of lyso phospholipids was beneficial to the maintenance of lipid homeostasis in *L. vannamei*. Pathway analysis showed that adding lysolecithin at 0.1% or 2% led to the same pathway changes, mainly affecting glycerophospholipid metabolism and cellular autophagy. The metabolic pathway of glycerophospholipids encompasses the whole process from synthesis to modification to degradation. Guo et al. [65] proposed that under certain conditions, the glycerophospholipid metabolic pathway is markedly enriched, which has substantial consequences for membrane function and signaling. The results indicate that the addition of lysolecithin significantly contributes to the maintenance of cell membranes, signaling, and substance transport through the glycerophospholipid metabolic pathway. Through the autophagy pathway, cells utilize the autophagy pathway to degrade lipid droplets, converting stored TGs and cholesterol esters into free fatty acids and glycerol for cellular utilization, a process crucial for regulating energy homeostasis and coping with nutritional deficiencies [66]. The addition of lysolecithin in the Ca^2+^/CaMKKβ/AMPK pathway activates AMPK, enhances autophagy by blocking the mammalian target of rapamycin (mTOR) pathway, and provides a protective effect and reduces apoptosis and damage through the autophagy mechanism [59].

Lecithin plays an essential role in forming cell membranes and are crucial for numerous functions within the cell. They contribute to the permeability barrier of cell membranes, providing the support matrix and surface for numerous catalytic processes, and participating in signaling in response to stimuli [67]. Our lipidomic analysis demonstrated a significant elevation in CL content within the hepatopancreas of shrimp administered a diet supplemented with lysolecithin. This observation is particularly noteworthy given that CL is a distinctive phospholipid predominantly localized in the inner mitochondrial membrane, where it plays a critical role in maintaining mitochondrial membrane architecture, facilitating electron transport chain function, and supporting energy production [68]. Although direct evidence in crustaceans remains sparse, research conducted in mammalian models and various fish species has indicated that lysolecithin supplementation can promote mitochondrial biogenesis and function. For example, studies in zebrafish have shown that lysolecithin enhances mitochondrial respiration rates [69]. Our findings of upregulated AMPK-CPT1 axis activity, a key regulator of mitochondrial fatty acid β-oxidation, further substantiate the hypothesis that dietary lysolecithin may augment mitochondrial metabolic efficiency in shrimp. Consequently, the observed increase in CL (DLCL, MLCL), LPA, PI and LPS, in conjunction with the activation of oxidative metabolism pathways, strongly implies that the enhanced growth and antioxidant capacity are likely supported by improved mitochondrial function. LPA and LPS are biologically active phospholipids that interact with specific G-protein-coupled receptors (GPCRs) to regulate macrophages. Increased LPA enhances inflammatory responses and immunomodulation [70]. LPS has been demonstrated to play a crucial role in processes such as cell migration, cell differentiation, and oxidative stress, and also induces mast cell degranulation through receptor interaction [71]. PA and PG were elevated at the 2% level of added lysolecithin in the results; PA inhibits mitochondrial fission, stimulates outer membrane fusion, and prevents excessive mitochondrial division [72]. Therefore, elevated levels of PA may facilitate the homeostasis of mitochondrial function in hepatopancreas. PG is a crucial component of cell membranes [73], influencing lipid metabolism and energy homeostasis [74]. Elevated levels of PG may improve membrane fluidity and stability, facilitate cell signaling and membrane-associated functions, and contribute to adaptation to environmental changes and stress response. Sphingolipids serve numerous physiological functions in organisms and are important components of cell membranes, participating in biological processes, including cell signaling, cell proliferation, apoptosis, and cell recognition [75]. GM3 is a simple ganglioglycolipid, which is essential in cell signaling, cell recognition, and cell adhesion [76], and elevated levels of GM3 may facilitate cell signaling and neurological development and regeneration. LSM is a sphingolipid containing choline phosphate, which is essential in the stability and fluidity of cell membranes [77], and elevated LSM levels may be related to cell membrane damage and repair, immune regulation, and inflammatory responses. SPHP is the major sphingolipid in the nervous system, which is involved in nerve myelin formation and maintenance [78], and elevated levels of SPHP may be related to the development and function of the nervous system, cellular signaling, and the development of the nervous system. Higher levels of SPHP may be related to the development and function of the nervous system, cell signaling, immune regulation, and inflammatory response. Elevated levels of Cer may induce lipotoxicity, inflammation, and metabolic disorders [79], and decreased levels of Cer may lead to a decrease in apoptosis and metabolic stability maintenance. SPH is an important sphingolipid metabolite, which is essential in cell signaling, cell growth, and apoptosis [80]. Elevated SPH at a 0.1% level of added lysolecithin may promote cell signaling, inflammatory response, and immune regulation. This experimental study demonstrates that among sphingolipids, GM3, LSM, and SPHP levels were elevated, and Cer, Hex1Cer, CerG2GNAc1, and CerG3GNAc1 levels were decreased, indicating that the addition of lysolecithin may affect hepatopancreatic neurodevelopment, immune regulation, cellular communication, and inflammatory response in *L. vannamei*.

Neutral lipids are water-insoluble but dissolve in organic solvents. They primarily include TG, CH, and ChE. Neutral lipids serve multiple functions in organisms, including energy storage, forming cell membrane structure, engaging in signaling, and modulating membrane fluidity [78]. This experimental study demonstrated that the addition of lysophospholipid neutral lipids maintained relative stability, which facilitated the equilibrium of energy metabolism, regulated the normal function of the immune system, and was essential for sustaining the structural integrity and function of cell membranes. Coenzymes are essential cofactors in many enzymatic reactions involved in energy metabolism, redox reactions, and other biochemical processes. This study demonstrated that the addition of lysolecithin elevated coenzymes, including Co(Q6), Co(Q7), Co(Q8), Co(Q9), and Co(Q10) and enhanced energy production capacity of the cells, which helpedmaintain the energy balance and cellular functions and facilitated the regulation of the metabolism and cellular signaling. Coenzyme Q10 (CoQ10), found in the inner mitochondrial membrane, plays key roles in the electron transport chain and ATP synthesis. Increasing CoQ10 enhances cellular antioxidant capacity and reduces cellular damage caused by oxidative stress [78,81]. Glyceroglycolipids are essential components of cell membranes and participate in various signaling pathways, significantly contributing to energy storage and metabolism [82]. MGMG is one of the simplest glyceroglycolipids with a monoacyl group and a galactose group, which serves as a precursor for more complex glyceroglycolipids; MGDG is derived from MGMG obtained through additional acylation, with one galactose moiety and two acyl groups; DGDG, which contains two galactose moieties and two acyl groups, represents a more complex glycerolipid [83]. In animal cells, similar glycolipids may contribute to maintaining the stability and functionality of cell membranes, although the specific role of DGDG has been less studied. This experimental study demonstrated that among glycerolipids, MGMG and MGDG decreased, and DGDG increased, indicating that the addition of lysolecithin may have converted MGMG and MGDG toward DGDG and that this conversion reflects a complex regulatory mechanism in the lipidome of cell membranes.

### 4.6. Limitations of the Study

This study has several limitations. The lipidomics analysis included only three groups and could benefit from a broader approach for deeper metabolic insights. The proposed Ca^2+^/CaMKKβ/AMPK pathway mechanism lacks direct evidence, as protein phosphorylation and functional validation experiments were not performed. No pre-test efficacy analysis was performed, but the sample size (*n* = 4 per group) is standard and sufficient for detecting statistical significance in key variables. Future research should expand lipidomics analysis, use methods like Western blot to assess phosphorylation of proteins such as AMPK, conduct gain- and loss-of-function experiments, and apply power analysis to determine optimal sample sizes.

## 5. Conclusions

This study found that adding 0.1% lysolecithin to the feed of *L. vannamei* achieved the best growth performance, improved lipid retention and fat digestibility as well as resistance to endoplasmic reticulum stress, while increasing tissue omega-3 fatty acids and promoting beneficial gene expression for lipid metabolism. The supplementation also protected hepatic tubules and maintained lipid homeostasis. Results indicate that 0.1% lysolecithin is optimal and can replace 2.0% lecithin in practical diets.

## Figures and Tables

**Table 1:** Formulation and chemical composition of experimental diets.

Ingredients % ^a^	DL2	RL0	RL0.1	RL0.5	RL1	RL1.5	RL2
Fish meal	25.00	25.00	25.00	25.00	25.00	25.00	25.00
Soybean meal	18.00	18.00	18.00	18.00	18.00	18.00	18.00
Wheat flour	21.99	23.99	23.89	23.49	22.99	22.49	21.99
Krill meal	5.00	5.00	5.00	5.00	5.00	5.00	5.00
Peanut meal	16.40	16.40	16.40	16.40	16.40	16.40	16.40
Brewer’s yeast	5.00	5.00	5.00	5.00	5.00	5.00	5.00
Fish oil	1.00	1.00	1.00	1.00	1.00	1.00	1.00
Soy oil	1.00	1.00	1.00	1.00	1.00	1.00	1.00
Mineral premix ^b^	1.00	1.00	1.00	1.00	1.00	1.00	1.00
Vitamin premix ^c^	1.00	1.00	1.00	1.00	1.00	1.00	1.00
Dicalcium phosphate	1.00	1.00	1.00	1.00	1.00	1.00	1.00
Choline chloride	0.50	0.50	0.50	0.50	0.50	0.50	0.50
Soy lecithin	2.00	0.00	0.00	0.00	0.00	0.00	0.00
Lysolecithin Soy lysolecithin	0.00	0.00	0.10	0.50	1.00	1.50	2.00
Vitamin C phosphate	0.10	0.10	0.10	0.10	0.10	0.10	0.10
Sodium alginate	1.00	1.00	1.00	1.00	1.00	1.00	1.00
Y_2_O_3_	0.01	0.01	0.01	0.01	0.01	0.01	0.01
Total	100.00	100.00	100.00	100.00	100.00	100.00	100.00
Calculated composition ^d^							
Crude fat, %	7.81	5.85	5.95	6.34	6.83	7.32	7.81
Crude protein, %	40.69	40.93	40.92	40.87	40.81	40.75	40.69
Crude ash, %	8.55	8.58	8.57	8.57	8.56	8.55	8.55

Note: ^a^ Fish meal, soybean meal, wheat flour, krill meal, peanut meal, brewer’s yeast, fish oil, dicalcium phosphate, choline chloride, Vitamin C-phosphate, and sodium alginate were purchased from Qingdao Baiwei Yingge Biotechnology Co., Ltd. (Qingdao, China). ^b^ A mineral premix was purchased from Guangzhou Xinghailisheng Biotechnology Co., Ltd. (Guangzhou, China). The composition of mineral premix was shown (g kg^−1^): KCl, 90; NaCl, 40; KI, 0.04; ZnSO_4_·7H_2_O, 4; CuSO_4_·5H_2_O, 3; CoSO_4_·7H_2_O, 0.02; MnSO_4_·H_2_O, 3; FeSO_4_·7H_2_O, 20; MgSO_4_·7H_2_O, 124; CaCO_3_, 215; Ca(H_2_PO_4_)_2_·2H_2_O, 500. ^c^ Vitamin premix was purchased from Guangzhou Bauxite Aquatic Technology Co., Ltd. (Guangzhou, China). Vitamin premix contains (g kg^−1^): Ve, 75; Vk, 2.5; VB_1_, 0.25; VB_2_, 1.0; VB_3_, 5.0; VB_6_, 0.75; VB_12_, 2.5; Va, 2.5; Vd, 6.25; VB_9_, 0.25; VB_h_, 379; VB_7_, 2.5. ^d^ The calculated composition was based on dry matter.

**Table 2:** Effects of dietary treatments on shrimp growth performance and nutrient digestibility (*n* = 4).

Items	DL2	RL0	RL0.1	RL0.5	RL1	RL1.5	RL2
Survival, %	97.50 ± 3.20	95.85 ± 4.20	100.00 ± 0.00	95.83 ± 5.00	99.18 ± 1.66	100.00 ± 0.00	97.50 ± 5.00
IBW ^1^	2.21 ± 0.06	2.18 ± 0.06	2.23 ± 0.05	2.21 ± 0.04	2.21 ± 0.04	2.16 ± 0.04	2.22 ± 0.09
FBW ^2^	10.70 ± 0.37	10.69 ± 0.28	11.25 ± 0.20	10.76 ± 0.52	10.80 ± 0.63	10.49 ± 0.56	10.68 ± 0.40
WG, %	385.70 ± 25.73	390.25 ± 1.10	404.51 ± 9.54	388.26 ± 30.30	387.82 ± 22.52	386.47 ± 25.89	381.45 ± 30.16
SGR	2.83 ± 0.08	2.82 ± 0.09	2.89 ± 0.04	2.83 ± 0.11	2.83 ± 0.08	2.82 ± 0.10	2.81 ± 0.11
FCR	1.46 ± 0.08	1.47 ± 0.03	1.46 ± 0.02	1.43 ± 0.06	1.44 ± 0.09	1.41 ± 0.03	1.44 ± 0.08
PR, %	125.55 ± 6.21	123.26 ± 4.41	125.69 ± 1.94	124.73 ± 6.10	125.81 ± 8.18	129.81 ± 2.19	129.94 ± 9.18
LR, %	50.07 ± 6.07 ^b^	56.01 ± 6.19 ^ab^	58.28 ± 2.16 ^ab^	62.83 ± 3.01 ^ab^	62.98 ± 3.21 ^ab^	67.24 ± 5.99 ^a^	66.69 ± 3.27 ^a^

Note: Different superscript letters within the same column mean significant differences between groups at *p* < 0.05. ^1^ Initial body weight (IBW, g); ^2^ Final body weight (FBW, g).

**Table 3:** Apparent digestibility coefficients (ADC, %) of crude protein and crude fat.

Items	DL2	RL0	RL0.1	RL0.5	RL1	RL1.5	RL2
Crude protein	72.13 ± 2.65	74.48 ± 0.19	71.59 ± 1.90	72.41 ± 1.87	74.36 ± 0.57	71.36 ± 1.03	70.76 ± 1.22
Crude fat	72.30 ± 1.36 ^d^	75.95 ± 1.77 ^bc^	76.32 ± 1.83 ^abc^	79.32 ± 0.07 ^a^	78.79 ± 0.21 ^ab^	76.07 ± 0.87 ^bc^	75.50 ± 0.47 ^cd^

Note: Different superscript letters within the same column mean significant differences between groups at *p* < 0.05.

**Table 4:** Nutritional composition of whole shrimp and muscle (% dry Weight).

Items	DL2	RL0	RL0.1	RL0.5	RL1	RL1.5	RL2
Composition of Whole Shrimp							
Moisture	77.53 ± 0.62	77.44 ± 0.53	77.59 ± 0.52	76.64 ± 0.55	77.15 ± 0.61	77.35 ± 0.30	77.41 ± 0.45
Crude Fat	4.43 ± 0.33 ^d^	4.62 ± 0.36 ^d^	5.00 ± 0.20 ^cd^	5.83 ± 0.31 ^bc^	6.24 ± 0.49 ^ab^	6.79 ± 0.58 ^ab^	6.97 ± 0.54 ^a^
Crude Protein	78.73 ± 0.84	78.12 ± 1.13	78.24 ± 0.18	77.33 ± 1.01	78.07 ± 0.75	78.05 ± 0.70	77.64 ± 1.08
Ash Content	12.70 ± 0.62	12.94 ± 1.17	12.22 ± 0.56	12.26 ± 0.86	12.21 ± 0.55	11.85 ± 0.73	12.75 ± 0.20
Muscle							
Moisture	75.91 ± 0.43	75.96 ± 0.21	76.42 ± 0.44	76.11 ± 0.56	76.2 ± 0.31	76.45 ± 0.32	75.99 ± 0.23
Crude Fat	3.43 ± 0.24 ^c^	3.49 ± 0.17 ^c^	3.72 ± 0.32 ^bc^	3.93 ± 0.23 ^abc^	4.09 ± 0.28 ^ab^	4.17 ± 0.04 ^ab^	4.29 ± 0.23 ^a^
Crude Protein	92.93 ± 0.26 ^a^	92.41 ± 0.24 ^a^	92.03 ± 0.45 ^ab^	91.82 ± 0.37 ^ab^	91.94 ± 0.42 ^ab^	91.95 ± 0.54 ^ab^	91.19 ± 0.56 ^b^
Ash Content	5.71 ± 0.51	5.52 ± 0.08	5.65 ± 0.27	5.37 ± 0.27	5.66 ± 0.24	5.69 ± 0.24	5.49 ± 0.17
Hepatopancreas							
Moisture	80.30 ± 0.82	81.96 ± 1.39	82.62 ± 2.14	81.05 ± 1.59	80.51 ± 0.43	81.81 ± 1.50	79.40 ± 0.89
Crude Fat	11.04 ± 1.99 ^d^	11.40 ± 0.46 ^cd^	10.38 ± 0.35 ^d^	12.17 ± 0.26 ^bcd^	15.03 ± 0.53 ^ab^	14.17 ± 0.18 ^abc^	15.59 ± 1.68 ^a^

Note: Data in the same column with different superscript letters differ significantly (*p* < 0.05). No pertinent measurements or analyses were performed regarding the moisture and ash content of the hepatopancreas.

**Table 5:** Effect of dietary treatments on antioxidant enzyme activity in the Hepatopancreas of *L. vannamei* (*n* = 4).

Items	DL2	RL0	RL0.1	RL0.5	RL1	RL1.5	RL2
T-AOC (U/mg protein)	3.73 ± 0.79	3.56 ± 0.38	3.10 ± 0.38	2.91 ± 0.71	3.66 ± 0.88	3.50 ± 0.53	2.55 ± 0.50
SOD (U/mg protein)	5.08 ± 0.13	5.01 ± 1.04	3.68 ± 0.43	5.54 ± 0.34	4.36 ± 2.01	5.38 ± 0.36	3.11 ± 0.98
GPx (U/mg protein)	255.70 ± 41.71	185.39 ± 36.25	195.39 ± 7.12	221.93 ± 23.53	244.82 ± 68.41	238.44 ± 36.36	240.53 ± 42.85
MDA (nmol/mg protein)	1.76 ± 0.52	1.19 ± 0.46	0.80 ± 0.30	1.65 ± 0.87	1.45 ± 0.40	2.44 ± 0.67	2.15 ± 1.01

**Table 6:** Effect of different dietary treatments on lipid metabolism enzymes in the hepatopancreas of *L. vannamei* (*n* = 4).

Items	DL2	RL0	RL0.1	RL0.5	RL1	RL1.5	RL2
TL (U/mg protein)	11.20 ± 1.42 ^a^	8.72 ± 1.44 ^a^	9.83 ± 0.44 ^a^	11.57 ± 1.94 ^a^	12.38 ± 3.41 ^a^	18.54 ± 3.52 ^b^	16.88 ± 2.68 ^b^
HL (U/mg protein)	5.13 ± 0.77 ^a^	3.65 ± 0.77 ^a^	4.65 ± 0.36 ^a^	5.33 ± 1.15 ^a^	5.77 ± 1.90 ^a^	8.15 ± 2.02 ^b^	7.81 ± 1.71 ^b^
LPL (U/mg protein)	6.06 ± 0.73 ^a^	5.07 ± 0.97 ^a^	5.18 ± 0.51 ^a^	6.24 ± 0.92 ^a^	6.60 ± 1.57 ^a^	10.39 ± 1.68 ^b^	9.07 ± 1.16 ^b^
Lipase (U/mg protein)	0.52 ± 0.09	0.70 ± 0.13	0.53 ± 0.11	0.65 ± 0.12	0.66 ± 0.12	0.66 ± 0.13	0.72 ± 0.19
ACC (U/L)	10.4 ± 0.18 ^a^	10.78 ± 0.19 ^ab^	10.98 ± 0.08 ^ab^	11.24 ± 0.32 ^b^	11.12 ± 0.01 ^b^	11.31 ± 0.63 ^b^	11.34 ± 0.46 ^b^
FAS (U/mL)	377.32 ± 26.74 ^a^	401.61 ± 37.92 ^ab^	420.73 ± 37.57 ^ab^	415.55 ± 26.13 ^ab^	399.58 ± 13.56 ^ab^	445.75 ± 34.45 ^b^	422.36 ± 19.01 ^ab^
CPT-1 (U/L)	78.23 ± 6.04	74.25 ± 8.15	78.56 ± 3.18	81.06 ± 0.85	80.42 ± 9.81	88.36 ± 8.70	84.11 ± 4.94
ATGL (U/mL)	72.64 ± 7.77	69.94 ± 5.82	70.08 ± 6.68	83.80 ± 22.26	83.37 ± 8.48	86.00 ± 21.13	81.9 ± 9.21

Note: Different lowercase letters in each column indicate that treatments differ significantly (*p* < 0.05).

## Data Availability

Data will be made available upon request.

## Supplementary Materials

The following supporting information can be downloaded at https://www.mdpi.com/article/10.3390/antiox14101209/s1, Figure S1: Pie charts illustrating the quantities and percentage distributions of the top 10 lipids in the hepatopancreas of *L. vannamei*; Table S1: Primers designed for Real-time PCR Amplification; Table S2: Effect of different dietary treatments on fatty acid profiles in the hepatopancreas of *L. vannamei* (*n* = 4); Table S3: Effect of different dietary treatments on fatty acid profiles in shrimp muscle (*n* = 4); Table S4: Comparison of lipid subclasses in the hepatopancreas of *L. vannamei* among different groups (*n* = 6).
