# Supplementary material for: Effects of Lysolecithin on Growth Performance, Antioxidant Capacity, and Lipid Metabolism of Litopenaeus vannamei
Source: Antioxidants (Basel). 2025 Oct 6;14(10):1209. doi: 10.3390/antiox14101209 (PMC12562013; doi:10.3390/antiox14101209)
Supplement: Supplementary file 1 [file antioxidants-14-01209-s001.zip › antioxidants-3825801-supplementary.pdf]

Table S1 Primers designed for Real-time PCR Amplification

cDNA	Forward Primer (5′–3′)	Reverse Primer (5′–3′)	Product size	GenBank No.
β-actin	CGAGGTATCCTCACCCTGAA	GTCATCTTCTCGCGGTTAGC	176	AF300705.2
Antioxidant-Related Genes				
Nrf1	TCCCAAGAGTGAGACAAAGATT	CACCAGTCTCAGAGTCGATA	86	XM_027363478.1
Nrf2	TCTTGTTGGTCCCTCGCTCCTC	TCACTGCTTGGGGTCATCCTTCC	89	XM_027367070.1
GPx	GGCACCAGGAGAACACTAC	CGACTTTGCCGAACATAAC	102	AY973252.2
SOD	GCAATGAATGCCCTTCTACC	CAGAGCCTTTCACTCCAACG	199	XM_027376216.1
CAT	TACTGCAAGTTCCATTACAAGACG	GTAATTCTTTGGATTGCGGTCA	285	XM_027383088.1
Hippo	TGAGCACAACCAAACCCACCATC	CATCGTCCGACTGTCCACTTCATC	86	MW415984.1
Endoplasmic Reticulum Stress-Related Genes				
IRE1	AGAAAGAAGGTGGCGACAGTTTG	TTGACGCCAGTCTCCTCGAA	80	XM_027370662.1
XBP1	GTGGATCAGCAGTATCCCAACC	TGCCAAGGCAGCTGTATTGA	113	XR_003477396.1
ATF6	CTGTTGGGACAAGGACCATAAGC	GAATTGTAGGTGTGGCAGCTGTTA	108	XM_027374810.1
ATF4	GAAGTCTGGAGCTGGAGCATCA	CAGGGACTCCAAAGGATGCTT	80	XM_027369231.1
Lipid Metabolism-Related Genes				
camkkβ	GAAACTTCCATTCCACGAT	TCACCCAGGGATGTTGCTT	182	XM_027368225.1
ampk	CTTTGCTGATGCTAATGCCT	TCCTTTGGGTAGTCCACGAT	187	KP272116.1
acc1	TGCATAGAAACGGCATTGCG	TTTGACACCTGAGCCAGACC	134	XM_027360190.1
SREBP	ACCATTGCCACTCCCCTA	GTTGCGTTTCTCGCCTTT	150	MG770374.1
fas	CGTGACACCCCTTCCTCAC	GAGAGTGTGAGGAACATAGACCA	196	HM595630.1
scd1	TGTCTTACACCTTATCAATGGC	CGTTCGTATGTTCCTCTTCGTC	154	XM_027374708.1
cd36	AACCAAGGTCCTGACCATCAC	AGGTGAGAGTCGACGAGGAA	210	XM_027374896.1
mcd	AAGACCACAGGAAGGGACCA	GACACTTGAGATGCCACCCA	114	XM_027376735.1
cpt1	ACTCCCGATAAGCACAC	TTCATACATCCACCCCCT	139	XM_027373671.1

Note: Nrf1 means nuclear respiratory factor 1; Nrf2 denotes nuclear factor erythroid 2-related factor 2; GPx denotes glutathione; SOD denotes superoxide dismutase; CAT denotes hydrogen peroxide; IRE1, inositol-requiring enzyme 1; XBP1, X-box binding protein 1; ATF6, activating transcription factor 6; ATF4, activating transcription factor 4; camkkβ, calmodulin-dependent protein kinase kinase; Ampk, 5'-AMP-activated protein kinase; acc1, acetyl-CoA carboxylase 1; SREBP, sterol regulatory element binding protein; fas, fatty acid synthase; scd1, stearoyl-CoA desaturase; cd36, cluster of differentiation 36; mcd, malonyl-CoA decarboxylase; cpt1, carnitine palmitoyl transferase 1.

Table S2. Effect of different dietary treatments on fatty acid profiles in the hepatopancreas of *L. vannamei* (n = 4).

Parameters	DL2	RL0	RL0.1	RL0.5	RL1	RL1.5	RL2
C14:0	0.57 ± 0.22^{b}	0.54 ± 0.17^{b}	0.36 ± 0.05^{ab}	0.42 ± 0.15^{ab}	0.23 ± 0.06^{a}	0.38 ± 0.17^{ab}	0.53 ± 0.27^{b}
C15:0	0.32 ± 0.02^{cd}	0.35 ± 0.02^{d}	0.30 ± 0.02^{bcd}	0.33 ± 0.04^{cd}	0.22 ± 0.02^{a}	0.27 ± 0.05^{b}	0.29 ± 0.02^{bc}
C16:0	17.93 ± 1.17^{ab}	18.99 ± 0.81^{b}	18.31 ± 1.05^{ab}	18.77 ± 1.49^{b}	16.50 ± 1.10^{a}	17.96 ± 1.56^{ab}	17.96 ± 0.73^{ab}
C17:0	0.74 ± 0.12^{ab}	0.79 ± 0.12^{ab}	0.79 ± 0.12^{ab}	0.75 ± 0.07^{ab}	0.86 ± 0.11^{b}	0.69 ± 0.13^{ab}	0.63 ± 0.12^{a}
C18:0	10.28 ± 2.42^{ab}	9.82 ± 1.93^{a}	11.01 ± 1.75^{ab}	10.09 ± 1.95^{ab}	13.15 ± 1.72^{b}	10.19 ± 2.27^{ab}	9.13 ± 1.55^{a}
C20:0	0.71 ± 0.07^{a}	0.76 ± 0.10^{ab}	0.75 ± 0.05^{ab}	0.73 ± 0.09^{a}	0.87 ± 0.06^{b}	0.82 ± 0.11^{ab}	0.78 ± 0.07^{ab}
C21:0	0.20 ± 0.03^{b}	0.16 ± 0.02^{a}	0.15 ± 0.02^{a}	0.15 ± 0.03^{a}	0.23 ± 0.02^{bc}	0.25 ± 0.03^{c}	0.25 ± 0.02^{c}
C22:0	0.71 ± 0.17	0.72 ± 0.11	0.81 ± 0.10	0.79 ± 0.19	0.88 ± 0.10	0.82 ± 0.14	0.80 ± 0.10
C23:0	0.26 ± 0.08^{ab}	0.19 ± 0.03^{a}	0.22 ± 0.02^{a}	0.19 ± 0.03^{a}	0.30 ± 0.06^{bc}	0.34 ± 0.04^{c}	0.35 ± 0.05^{c}
C24:0	0.57 ± 0.11	0.57 ± 0.17	0.66 ± 0.14	0.65 ± 0.07	0.50 ± 0.09	0.52 ± 0.32	0.49 ± 0.10
SFA	32.30 ± 1.36^{ab}	32.88 ± 1.41^{ab}	33.35 ± 0.82^{ab}	32.88 ± 0.73^{ab}	33.72 ± 2.00^{b}	32.23 ± 1.29^{ab}	31.21 ± 1.54^{a}
C16:1	1.16 ± 0.31	1.47 ± 0.39	1.08 ± 0.06	1.20 ± 0.29	0.98 ± 0.11	1.26 ± 0.42	1.52 ± 0.58
C18:1n9c	16.59 ± 2.91	17.15 ± 2.32	15.69 ± 1.98	16.95 ± 2.30	15.76 ± 0.70	17.82 ± 2.40	17.76 ± 2.14
C20:1	3.03 ± 0.15^{a}	3.69 ± 0.15^{bc}	3.47 ± 0.20^{b}	3.66 ± 0.26^{b}	3.99 ± 0.18^{c}	3.75 ± 0.18^{bc}	3.56 ± 0.22^{b}
C24:1	0.88 ± 0.17^{a}	0.92 ± 0.14^{ab}	1.00 ± 0.06^{ab}	0.96 ± 0.25^{ab}	1.14 ± 0.12^{c}	1.03 ± 0.10^{ab}	1.06 ± 0.15^{ab}
MUFA	21.65 ± 3.17	23.23 ± 2.55	21.24 ± 2.00	22.76 ± 2.62	21.87 ± 0.61	23.86 ± 2.79	23.90 ± 2.35
C18:3n3	0.71 ± 0.19^{c}	0.39 ± 0.11^{ab}	0.29 ± 0.06^{a}	0.37 ± 0.05^{ab}	0.38 ± 0.08^{ab}	0.61 ± 0.18^{bc}	0.78 ± 0.30^{c}
C20:3n3	0.29 ± 0.05^{ab}	0.32 ± 0.07^{ab}	0.35 ± 0.05^{b}	0.31 ± 0.07^{ab}	0.26 ± 0.09^{ab}	0.26 ± 0.04^{ab}	0.24 ± 0.03^{a}
C20:5n3	8.89 ± 1.83^{abc}	10.21 ± 1.64^{bc}	11.18 ± 2.17^{c}	9.58 ± 1.64^{abc}	10.04 ± 0.88^{bc}	7.86 ± 2.25^{ab}	7.02 ± 1.08^{a}
C22:6n3	8.87 ± 0.94^{ab}	10.34 ± 0.47^{cd}	10.82 ± 0.58^{d}	10.13 ± 0.83^{bcd}	9.08 ± 1.21^{abc}	8.11 ± 1.26^{a}	8.04 ± 0.71^{a}
∑n-3 PUFA	18.76 ± 2.65^{abc}	21.26 ± 1.99^{c}	22.65 ± 2.65^{c}	20.39 ± 2.30^{bc}	19.77 ± 2.09^{abc}	16.84 ± 3.32^{ab}	16.07 ± 1.50^{a}
C18:2n6c	17.22 ± 2.72^{b}	12.23 ± 1.91^{a}	11.26 ± 1.08^{a}	12.16 ± 1.29^{a}	12.17 ± 1.27^{a}	15.55 ± 2.63^{b}	16.88 ± 2.24^{b}
C20:3n6	0.06 ± 0.02^{a}	0.11 ± 0.03^{ab}	0.13 ± 0.06^{b}	0.16 ± 0.02^{b}	0.14 ± 0.05^{b}	0.14 ± 0.06^{b}	0.11 ± 0.04^{ab}
C20:4n6	3.95 ± 0.38^{a}	4.41 ± 0.11^{ab}	4.15 ± 0.12^{ab}	4.10 ± 0.51^{ab}	4.29 ± 0.47^{ab}	4.50 ± 0.26^{b}	4.55 ± 0.20^{b}
∑n-6 PUFA	21.23 ± 2.96^{b}	16.75 ± 1.99^{a}	15.54 ± 0.93^{a}	16.42 ± 1.65^{a}	16.61 ± 1.44^{a}	20.19 ± 2.71^{b}	21.54 ± 2.46^{b}
C20:2	3.13 ± 0.25^{ab}	2.66 ± 0.21^{a}	3.29 ± 0.27^{b}	3.34 ± 0.25^{b}	3.62 ± 0.39^{b}	3.31 ± 0.46^{b}	3.02 ± 0.59^{ab}
C22:1n9	2.86 ± 2.12	3.13 ± 1.40	3.81 ± 0.95	4.10 ± 1.26	4.27 ± 1.59	3.48 ± 1.33	4.15 ± 1.85
C22:2	0.08 ± 0.05^{a}	0.09 ± 0.04^{ab}	0.12 ± 0.05^{ab}	0.11 ± 0.04^{ab}	0.15 ± 0.03^{b}	0.10 ± 0.03^{ab}	0.11 ± 0.03^{ab}
PUFA	6.07 ± 2.36	5.88 ± 1.41	7.23 ± 0.78	7.55 ± 1.38	8.04 ± 1.43	6.89 ± 1.61	7.28 ± 1.71
∑n-3/∑n-6PUFA	0.91 ± 0.28^{abc}	1.29 ± 0.27^{d}	1.47 ± 0.24^{d}	1.26 ± 0.27^{cd}	1.19 ± 0.12^{bcd}	0.86 ± 0.28^{ab}	0.76 ± 0.16^{a}

Note: Different superscript letters within the same column mean a significant difference between groups (p < 0.05). SFA, saturated fatty acid; MUFA, monounsaturated fatty acid; n−3 PUFA, n−3 polyunsaturated fatty acid; n−6 PUFA, n−6 polyunsaturated fatty acid; PUFA, polyunsaturated fatty acid.

Table S3. Effect of different dietary treatments on fatty acid profiles in shrimp muscle (*n* = 4).

Items	DL2	RL0	RL0.1	RL0.5	RL1	RL1.5	RL2
C14:0	0.21 ± 0.08^{ab}	0.22 ± 0.07^{ab}	0.17 ± 0.06^{a}	0.21 ± 0.02^{ab}	0.27 ± 0.09^{ab}	0.24 ± 0.04^{ab}	0.28 ± 0.02^{b}
C15:0	0.18 ± 0.02	0.18 ± 0.04	0.17 ± 0.05	0.18 ± 0.01	0.20 ± 0.04	0.23 ± 0.07	0.23 ± 0.01
C16:0	20.82 ± 1.26	19.79 ± 2.47	20.26 ± 2.87	19.97 ± 0.40	21.06 ± 0.59	21.20 ± 0.78	21.03 ± 0.30
C17:0	0.98 ± 0.13	0.99 ± 0.04	0.99 ± 0.03	0.98 ± 0.04	0.95 ± 0.02	0.95 ± 0.02	0.94 ± 0.02
C18:0	16.77 ± 3.62	15.61 ± 0.72	15.33 ± 0.61	15.17 ± 0.76	15.11 ± 0.46	14.71 ± 0.31	14.72 ± 0.16
C20:0	1.40 ± 0.36	1.39 ± 0.09	1.30 ± 0.12	1.25 ± 0.05	1.22 ± 0.07	1.15 ± 0.08	1.28 ± 0.08
C21:0	0.25 ± 0.07^{b}	0.19 ± 0.03^{a}	0.18 ± 0.02^{a}	0.18 ± 0.01^{a}	0.19 ± 0.02^{ab}	0.19 ± 0.03^{ab}	0.21 ± 0.04^{ab}
C22:0	1.38 ± 0.31	1.24 ± 0.17	1.22 ± 0.22	1.32 ± 0.19	1.14 ± 0.12	1.12 ± 0.15	1.30 ± 0.06
C23:0	0.36 ± 0.12	0.28 ± 0.04	0.30 ± 0.06	0.29 ± 0.04	0.30 ± 0.05	0.28 ± 0.02	0.32 ± 0.05
C24:0	0.58 ± 0.22	0.50 ± 0.10	0.53 ± 0.10	0.54 ± 0.06	0.54 ± 0.08	0.51 ± 0.12	0.53 ± 0.03
SFA	42.91 ± 3.82	40.38 ± 1.61	40.45 ± 2.10	40.08 ± 0.76	40.95 ± 0.69	40.59 ± 1.07	40.85 ± 0.27
C16:1	0.80 ± 0.05^{a}	1.11 ± 0.17^{b}	1.00 ± 0.22^{ab}	1.17 ± 0.10^{b}	1.13 ± 0.15^{b}	0.98 ± 0.06^{ab}	0.86 ± 0.05^{a}
C18:1n9	11.62 ± 1.53^{ab}	12.95 ± 0.45^{c}	12.58 ± 0.86^{bc}	13.65 ± 0.51^{c}	12.40 ± 0.38^{abc}	12.76 ± 0.57^{bc}	11.29 ± 0.57^{a}
C20:1	1.25 ± 0.40^{ab}	1.47 ± 0.16^{b}	1.31 ± 0.12^{ab}	1.46 ± 0.11^{b}	1.28 ± 0.09^{ab}	1.19 ± 0.02^{ab}	1.16 ± 0.03^{a}
C24:1	0.58 ± 0.15	0.64 ± 0.15	0.63 ± 0.11	0.66 ± 0.14	0.57 ± 0.07	0.55 ± 0.07	0.62 ± 0.03
MUFA	14.25 ± 1.99^{ab}	16.15 ± 0.58^{c}	15.54 ± 1.03^{bc}	16.94 ± 0.67^{c}	15.39 ± 0.18^{abc}	15.47 ± 0.57^{bc}	13.93 ± 0.54^{a}
C18:3n3	0.57 ± 0.13^{b}	0.42 ± 0.02^{a}	0.39 ± 0.04^{a}	0.47 ± 0.05^{a}	0.57 ± 0.06^{b}	0.58 ± 0.06^{b}	0.60 ± 0.03^{b}
C20:3n3	0.18 ± 0.02	0.16 ± 0.02	0.17 ± 0.02	0.16 ± 0.01	0.17 ± 0.02	0.17 ± 0.03	0.17 ± 0.03
C20:5n3	11.24 ± 2.47^{a}	13.13 ± 0.21^{b}	13.22 ± 0.37^{b}	12.04 ± 0.22^{ab}	12.23 ± 0.37^{ab}	11.90 ± 0.28^{ab}	12.26 ± 0.44^{ab}
C22:6n3	10.02 ± 2.46^{a}	11.83 ± 0.63^{b}	11.66 ± 0.26^{ab}	11.10 ± 0.36^{ab}	10.87 ± 0.69^{ab}	10.93 ± 0.29^{ab}	11.29 ± 0.41^{ab}
∑n-3 PUFA	22.01 ± 5.02^{a}	25.53 ± 0.70^{b}	25.44 ± 0.18^{b}	23.77 ± 0.20^{ab}	23.83 ± 0.89^{ab}	23.58 ± 0.25^{ab}	24.32 ± 0.80^{ab}
C18:2n6	13.48 ± 1.04^{b}	11.15 ± 0.34^{a}	10.89 ± 0.48^{a}	11.68 ± 0.451^{a}	12.99 ± 0.66^{b}	13.39 ± 0.96^{b}	13.69 ± 0.35^{b}
C20:3n6	0.12 ± 0.08^{bc}	0.06 ± 0.01^{ab}	0.06 ± 0.01^{ab}	0.06 ± 0.01^{ab}	0.05 ± 0.01^{a}	0.09 ± 0.06^{abc}	0.13 ± 0.02^{c}
C20:4n6	1.68 ± 0.17^{a}	2.12 ± 0.17^{c}	2.00 ± 0.17^{bc}	2.01 ± 0.140^{bc}	1.88 ± 0.09^{b}	1.74 ± 0.04^{a}	1.82 ± 0.11^{ab}
∑n-6 PUFA	15.25 ± 1.22^{b}	13.33 ± 0.37^{a}	12.95 ± 0.55^{a}	13.74 ± 0.48^{a}	14.92 ± 0.71^{b}	15.22 ± 1.01^{b}	15.64 ± 0.44^{b}
C20:2	1.84 ± 0.07^{b}	1.52 ± 0.11^{a}	1.55 ± 0.19^{ab}	1.60 ± 0.05^{ab}	1.62 ± 0.14^{ab}	1.74 ± 0.16^{ab}	1.44 ± 0.39^{a}
C22:1n9	3.64 ± 0.98	3.01 ± 1.14	3.99 ± 2.73	3.77 ± 1.34	3.22 ± 1.85	3.33 ± 0.59	3.71 ± 1.35
C22:2	0.10 ± 0.03	0.08 ± 0.02	0.09 ± 0.07	0.10 ± 0.04	0.07 ± 0.04	0.08 ± 0.02	0.10 ± 0.01
PUFA	5.58 ± 0.97	4.60 ± 1.21	5.63 ± 2.95	5.47 ± 1.37	4.92 ± 1.84	5.15 ± 0.64	5.25 ± 1.32
∑n-3/∑n-6PUFA	1.43 ± 0.24^{a}	1.92 ± 0.10^{c}	1.97 ± 0.09^{c}	1.73 ± 0.05^{b}	1.60 ± 0.09^{ab}	1.56 ± 0.10^{ab}	1.56 ± 0.05^{ab}

Note: Different lowercase letters within the same column indicate significant differences among treatments ($p < 0.05$). SFA, saturated fatty acid; MUFA, monounsaturated fatty acid; n–3 PUFA, n–3 polyunsaturated fatty acid; n–6 PUFA, n–6 polyunsaturated fatty acid; PUFA, polyunsaturated fatty acid.

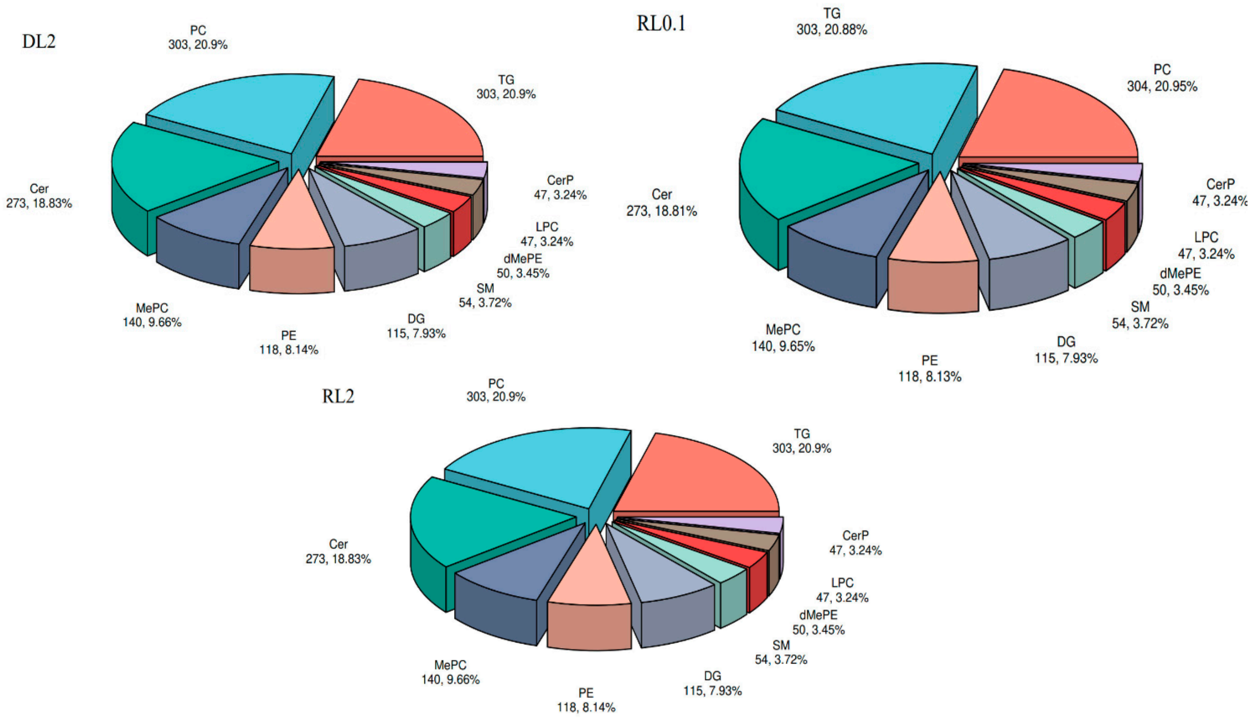

Figure S1. Pie charts illustrating the quantities and percentage distributions of the top 10 lipids in the hepatopancreas of *L. vannamei*.

Table S4. Comparison of lipid subclasses in the hepatopancreas of *L. vannamei* among different groups (n = 6).

Group	ClassKey	Lipid Name	Fold change (RL0.1/DL2)	Fold change (RL2/DL2)
	PI	Phosphatidylinositol	9.81	/
	LPS	Lyso-phosphatidylserine	3.82	/
Phospholipids	LPA	Lyso-phosphatidic acid	2.69	0.61
	DLCL	Cardiolipin	2.51	/
	MLCL	Cardiolipin	2.43	/
	CL	Cardiolipin	2.38	/
	PA	Phosphatidic acid	/	1.67
	LPE	Lyso-phosphatidylethanolamine	0.45	/
	PE	Phosphatidylethanolamine	0.66	/
	LPG	Lyso-phosphatidylglycerol	0.51	/
	PG	Phosphatidylglycerol	/	1.81
Sphingolipids	CerPE	Ceramide phosphoethanolamines	0.62	0.53
	Hex1SPH	Glucosylsphingosine	1.69	/
	Hex1Cer	Simple Glc series	0.66	/
	CerG2GNAc1	Simple Glc series	0.37	0.51
	CerG3GNAc1	Simple Glc series	0.62	/
	CerG3GNAc2	Simple Glc series	3.62	1.91
	GM3	Gangliosides	7.21	1.62
	LSM	Lysosphingomyelin	9.24	6.52
	SPH	Sphingosine	1.67	/
	SPHP	Sphingosine phosphate	81.86	5.11
Neutral lipids	ChE	Cholesterol Ester	1.68	/
	TG	Triglyceride	1.60	/
	ZyE	Zymosterol Ester	1.86	/
Fatty acyl and other lipids	Co(Q6)	Coenzyme	3.44	2.82
	Co(Q7)	Coenzyme	3.01	2.51
	Co(Q8)	Coenzyme	3.9	3.88
	Co(Q9)	Coenzyme	1.78	2.14
	Co(Q10)	Coenzyme	3.06	2.42
	LPEt	Lyso-phosphatidylethanol	0.24	0.10
Glycoglycerolipids (plants)	MGMG	Monogalactosylmonoacylglycerol	0.27	0.51
	MGDG	Monogalactosyldiacylglycerol	0.41	0.60
	DGDG	Digalactosyldiacylglycerol	2.50	
	SQDG	Sulfoquinovosyldiacylglycerol	0.23	0.38
Derivatized lipids (biotinylation, diazomethane)	BiotinylPE	Glycerophosphoethanolamine-n-(biotinyl)	2.33	2.17
	BisMePA	Bis-methyl phosphatidic acid	0.64	/
Fatty acyl carnitines	CarE	Carnitine Esters	0.34	0.38
